# Cryptic Diversity, but to What Extent? Discordance Between Single-Locus Species Delimitation Methods Within Mainland Anoles (Squamata: Dactyloidae) of Northern Central America

**DOI:** 10.3389/fgene.2019.00011

**Published:** 2019-02-11

**Authors:** Erich P. Hofmann, Kirsten E. Nicholson, Ileana R. Luque-Montes, Gunther Köhler, César A. Cerrato-Mendoza, Melissa Medina-Flores, Larry David Wilson, Josiah H. Townsend

**Affiliations:** ^1^Department of Biology, Indiana University of Pennsylvania, Indiana, PA, United States; ^2^Department of Biological Sciences, Clemson University, Clemson, SC, United States; ^3^Department of Biology, Central Michigan University, Mount Pleasant, MI, United States; ^4^Senckenberg Forschungsinstitut und Naturmuseum, Frankfurt, Germany; ^5^Federación Hondureña de Deportes de Montaña Y Escalada, Departamento de Francisco Morazán, Tegucigalpa, Honduras; ^6^Centro Zamorano de Biodiversidad, Escuela Agrícola Panamericana Zamorano, Departamento de Francisco Morazán, Tegucigalpa, Honduras

**Keywords:** ABGD, *Anolis*, cytochrome c oxidase subunit I (COI), DNA barcoding, GMYC, *Norops*, PTP, RESL

## Abstract

Single-locus molecular barcoding is a useful method for identifying overlooked and undescribed biodiversity, providing the groundwork for further systematic study and taxonomic investigation. A variety of methods for delimiting species from barcoding libraries have been developed and applied, allowing for rapid estimates of species diversity in a broad range of taxa. However, tree-based and distance-based analyses can infer different group assignments, potentially over- or underestimating the number of putative species groups. Here, we explore diversity of mainland species of anole lizards from the Chortís Block biogeographical province of northern Central America using a DNA barcoding approach, generating and analyzing cytochrome oxidase subunit I (COI) sequences for over 400 samples assignable to 33 of 38 (86.8%) native and one introduced mainland species. We subsequently tested the effects different models of nucleotide substitution, different species-delimitation algorithms, and reducing our dataset had on species delimitation estimates. We performed of two distance-based (ABGD, RESL) and three tree-based (bPTP, mPTP, GMYC) analyses on both the full dataset and a dataset consisting only of unique halotypes. From 34 nominal taxa, analyses of the full dataset recovered between 34 and 64 operational taxonomic units (OTUs), while analyses of the reduced dataset inferred between 36 and 59. Reassigning individuals to either mPTP-inferred or ABGD clustered (7.2% threshold) groups improved the detection of a barcoding gap across three different models of nucleotide substitution, removing overlap between intra- and interspecific distances. Our results highlight the underestimated diversity of mainland Chortís Block anoles, but the lack of congruence between analyses demonstrates the importance of considering multiple analytical methods when dealing with single-locus datasets. We recommend future studies consider the effects of different models of nucleotide substitution on proposed barcoding gaps, as well as the effect reducing a dataset to unique haplotypes may have on proposed diversity estimates.

## Introduction

The majority of fields in the biological sciences rely on the proper identification of species (Wheeler, 2004; de Queiroz, 2007); thus, the efficient and reliable identification and delimitation of taxa is of pivotal importance. A valuable tool for characterizing the taxonomic diversity of large clades of organisms with phenotypically conserved taxa both quickly and efficiently is DNA barcoding (Meyer and Paulay, 2005). The use of an approximately 650 basepair (bp) sequence of mitochondrial DNA (mtDNA) from the cytochrome c oxidase subunit I (COI) gene has been recognized as an effective “barcode” for animals (Hebert et al., 2003), though other fragments can be effective for species identification also (Hajibabaei et al., 2006). This target region can be sequenced easily from an unknown specimen and referenced against a library of known taxa to discern whether the specimen falls out within a recognized lineage or not (Meyer and Paulay, 2005).

Accelerating the pace of species discovery is a secondary goal of DNA barcoding (Stoeckle, 2003; Hebert et al., 2004), and the discovery of cryptic lineages using single-locus delimitation analyses can have profound impacts on a range of fields (Bickford et al., 2007). This approach can highlight areas where more careful taxonomic study, integrating morphological examination with multi-locus molecular analyses, is needed (Hajibabaei et al., 2007; Padial et al., 2009, 2010), especially in areas where genetic diversity is masked by conserved morphology (Hebert et al., 2003). Single-locus barcoding analyses, however, do not provide enough information to formally describe new species (DeSalle, 2006). Discordance between the boundaries of putative species inferred by different methods of single-locus delimitation can lead to uncertainty in diversity inferences, due to either over- or under-estimating the true number of lineages present in a sample (Blair and Bryson, 2017).

DNA barcoding has been applied to numerous vertebrate (e.g., Hebert et al., 2004; Álvarez-Castañeda et al., 2011; Mendoza et al., 2016; Ramirez et al., 2017; Barman et al., 2018) and invertebrate (e.g., Barrett and Hebert, 2005; Moura et al., 2011; Barco et al., 2016; Jin et al., 2018) taxa, but the barcoding of herpetofauna largely has lagged behind. The initiation of the “ColdCode” campaign (Murphy et al., 2013) has led to a more concentrated effort to provide reference libraries for reptiles and amphibians, including those focused on specific taxa (e.g., Dang et al., 2015; Liu et al., 2015) and broad-scale regional assessments (e.g., Nagy et al., 2012; Guarnizo et al., 2015; Hawlitscheck et al., 2016; Vasconcelos et al., 2016; Deichmann et al., 2017).

**FIGURE 1:**
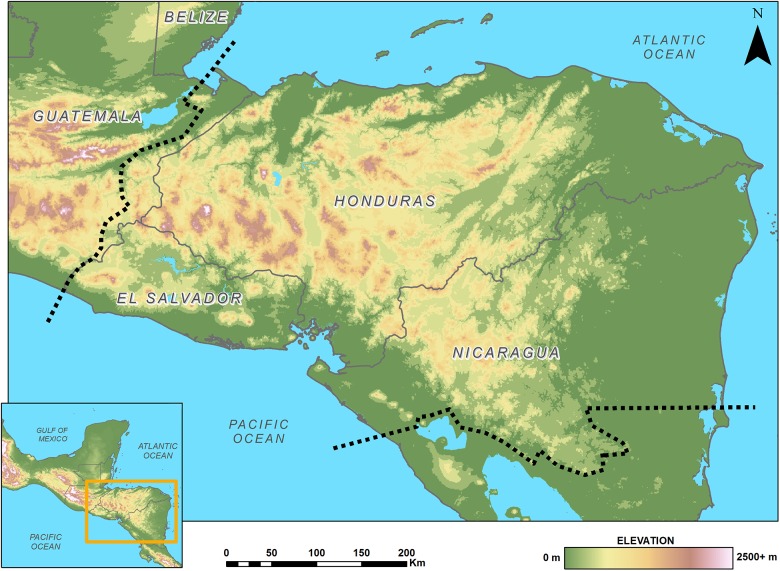
Map of the Chortís Block Biogeographical Province; boundaries are denoted by a dashed line.

In Northern Central America, the Chortís Block biogeographic province is increasingly being recognized as an important and distinctive area for herpetofaunal diversity (Figure 1; Townsend, 2014). This geologically and ecologically-delimited region, also referred to as “Eastern Nuclear Central America” (Campbell, 1999; Wilson and Townsend, 2007; Townsend, 2009), is comprised of eastern Guatemala, all of mainland Honduras and El Salvador, and northern Nicaragua, including Isla del Tigre, the Honduran Islas de la Bahía, and the Nicaraguan Cayos Miskitos (Townsend, 2014). Over 400 reptile and amphibian species are found here, and importantly, over 37% of those recognized species are endemic to the region, including more than half of the area’s amphibians and over 30% percent of its squamates (Townsend, 2014).

Anoles (Squamata: Dactyloidae) are particularly strong representatives of the Chortís Block’s squamate diversity, the region’s high levels of endemism, and the need for increased study and conservation work. As currently considered, thirty-eight species of anole lizards are native to the mainland Chortís Block, 20 (52.6%) of which are considered endemic (Table 1; Townsend, 2014; McCranie and Köhler, 2015; Köhler et al., 2016; Hofmann and Townsend, 2017, 2018). Two additional species, *Anolis allisoni* and *Norops sagrei*, have been introduced to the northern coast of mainland Honduras (McCranie and Köhler, 2015). They inhabit a variety of physiographic regions, habitats, and ecological roles throughout the Chortís Block (McCranie and Köhler, 2015); however, their often-conserved morphology combined with the paucity of published data on these species has led to a multitude of taxonomic complications.

**Table 1:** Summary of the mainland anoles of the Chortís Block: Species, number of samples (*N*) and localities used in this paper, and conservation scores.

Species	*N*	Sampling localities (COUNTRY: Department)	IUCN (2017)	Townsend and Wilson, 2010	McCranie and Köhler, 2015	EVS Category (Johnson et al., 2015)
*Anolis allisoni*	2	HONDURAS: Islas de la Bahia	–	LC	LC	M
*Norops amplisquamosus*	6	HONDURAS: Cortés	E	E	CE	H
*Norops beckeri*	-	–	–	–	LC	M
*Norops biporcatus*	4	HONDURAS: Cortés; NICARAGUA: Jinotega	–	LC	LC	L
*Norops caceresae*	21	HONDURAS: Intibucá, La Paz	–	LC^1^	LC^1^	L^1^
*Norops capito*	8	HONDURAS: Olancho; NICARAGUA: Atlántico Norte, Jinotega, Matagalpa	–	LC	LC	M
*Norops carpenteri*	-	–	–	–	LC	H
*Norops crassulus*	1	EL SALVADOR: Santa Ana	–	LC	LC	L
*Norops cupreus*	16	HONDURAS: Colon, Olancho; NICARAGUA: Atlántico Norte, Jinotega	–	LC	LC	M
*Norops cusuco*	5	HONDURAS: Cortés	E	E	E	H
*Norops heteropholidotus*	36	EL SALVADOR: Chalatenango; HONDURAS: Intibucá, Lempira, Ocotepeque	–	E	LC	H
*Norops johnmeyeri*	7	HONDURAS: Cortés	–	E	NT	H
*Norops kreutzi*	3	HONDURAS: Atlántida, Yoro	–	CE	E	H
*Norops laeviventris*	19	HONDURAS: Comayagua, Francisco Morazán, Intibucá, Lempira, Olancho, Yoro; NICARAGUA: Jinotega, Matagalpa	–	LC	LC	L
*Norops lemurinus*	22	HONDURAS: Atlántida, Colon, Cortés, Islas de la Bahia, Santa Bárbara	–	LC	LC	L
*Norops limifrons*	11	HONDURAS: Olancho; NICARAGUA: Jinotega	–	LC	LC	H
*Norops loveridgei*	7	HONDURAS: Atlántida	E	E	NT	H
*Norops macrophallus*	-	–	–	LC^2^	–	H
*Norops mccraniei*	40	HONDURAS: Comayagua, Cortés, Francisco Morazán, Lempira, Olancho, Santa Bárbara, Yoro	–	LC	LC	M
*Norops morazani*	54	HONDURAS: Francisco Morazán	–	CE	V	H
*Norops muralla*	-	–	V	CE	V	H
*Norops ocelloscapularis*	1	HONDURAS: Copán	–	E	NT	H
*Norops oxylophus*	3	NICARAGUA: Atlántico Norte	–	LC	LC	M^4^
*Norops petersii*	1	HONDURAS: Cortés	–	V	LC	L
*Norops pijolense*	14	HONDURAS: Yoro	–	E	V	H
*Norops purpurgularis*	1	HONDURAS: Yoro	–	E	V	H
*Norops quaggulus*	3	NICARAGUA: Jinotega, Matagalpa	–	LC	LC	H
*Norops rodriguezii*	7	HONDURAS: Cortés, Lempira, Santa Bárbara	–	LC	LC	M
*Norops rubribarbaris*	7	HONDURAS: Atlántida, La Paz, Santa Bárbara	–	E	V	H
*Norops serranoi*	1	EL SALVADOR: La Libertad	–	LC^2^	–	M
*Norops sminthus*	14	HONDURAS: Comayagua, Olancho	DD	E	LC	H
*Norops uniformis*	5	MEXICO: Chiapas, Veracruz	–	LC	LC	M
*Norops unilobatus*	3	HONDURAS: Cortés, Santa Bárbara	–	–	LC	L
*Norops wampuensis*	-	–	–	E	CE	H
*Norops wellbornae*	10	HONDURAS: Valle	–	–	LC	H
*Norops wermuthi*	2	NICARAGUA: Jinotega	–	V^3^	–	H
*Norops wilsoni*	4	HONDURAS: Atlántida	–	–	–	–
*Norops yoroensis*	59	HONDURAS: Atlántida, Cortés, Francisco Morazán, Yoro	–	E	NT	H
*Norops zeus*	15	HONDURAS: Atlántida, Cortés, Yoro	–	E	LC	H


*For complete sampling data, see Supplementary Table 1. Abbreviations: –, not analyzed; DD, Data Deficient; LC, Least Concern; V, Vulnerable; NT, Near Threatened; E, Endangered; CE, Critically Endangered; L, Low; M, Medium; H, High. ^*1*^As Norops crassulus (in part; see Hofmann and Townsend, 2018). ^*2*^Greenbaum and Komar (2010). ^*3*^Sunyer and Köhler (2010). ^*4*^McCranie and Köhler (2015).*

Here, we highlight the discordance between putative species limits inferred by several popular single-locus delimitation analyses. To this end, we test two distance-based and three tree-based methods on a DNA barcode library for anoles of the mainland Chortís Block of Northern Central America—consisting of sequence data for 33 of the 38 (86.8%) native species and one introduced to the mainland (otherwise native to Cuba and to the Islas de la Bahía, as well as numerous other islands and keys in the Caribbean). Our goals are to provide an efficient molecular reference for species identification and draw attention to cryptic lineages in need of further taxonomic investigation, while simultaneously stressing the importance of incorporating multiple tests before drawing conclusions of cryptic species-level diversity.

## Materials and Methods

### Justification of Nomenclature

We follow Nicholson et al. (2018) in their application of a rank-based, multi-genera taxonomy for anoles (valid under the International Code of Zoological Nomenclature; ICZN) based on the clade names proposed by Poe et al. (2017). As such, we refer to the monophyletic grouping of beta anoles as *Norops* (sensu Nicholson et al., 2018), while recognizing the criticisms of the multiple-genera taxonomy (e.g., Poe, 2013). All native, mainland *Norops* in the Chortís Block are beta anoles and members of clade *Draconura* [Poe et al., 2017 (*auratus* group of Nicholson et al., 2012)], one of the three clades within *Norops*, all of which share the synapomorphy of anterolaterally directed transverse processes on their caudal vertebrae (Etheridge, 1959; Nicholson, 2002) as well as numerous molecular characters (Nicholson et al., 2012; Poe et al., 2017).

### Field Sampling

Between June 2006 and January 2016, we sampled 96 localities in the Chortís Block, including localities within 17 of 18 departments in Honduras and four of 15 departments in northern Nicaragua (Table 1 and Supplementary Table 1). Tissue samples were collected from vouchers and stored in SED buffer (250 mM EDTA/20% DMSO/saturated NaCl; Seutin et al., 1991; Williams, 2007). Voucher specimens were preserved in 10% formalin solution, and later transferred to 70% ethanol for permanent storage. Vouchers were deposited in the Florida Museum of Natural History (FLMNH), University of Florida (UF); the Museum of Vertebrate Zoology, Berkeley (MVZ); the National Museum of Natural History, Smithsonian Institution (USNM); the Senckenberg Forschungsinstitut und Naturmuseum (SMF); and the natural history collection of the Universidad Nacional Autónoma de Nicaraga-León (UNAN). For one taxon where samples from within the Chortís Block were not available (*Norops uniformis*) and another with uncertain species boundaries (*N. cupreus*), samples from outside the Chortís Block were included (Table 1). Three additional samples representing populations of three species from El Salvador were received from the Herpetology collections of the University of Kansas Biodiversity Institute (KU). All specimens were identified *a priori* based on external morphology, using the keys of Köhler (2008) or McCranie and Köhler (2015). We follow McCranie and Köhler (2015) in considering *Norops dariense* a synonym of *Norops cupreus*, until further taxonomic study is completed.

### COI Amplification and Sequencing

DNA extraction, amplification, and sequencing prior to August 2015 was carried out at the Smithsonian Institution Laboratory of Analytical Biology (Suitland, MD, United States) following standardized DNA Barcode of Life (BOLD) protocols, while all other sequences were generated in the Townsend Lab at the Indiana University of Pennsylvania (Indiana, PA, United States). Whole-genome DNA was extracted from tissue using PureLink Genomic DNA Kits (Life Technologies). The mitochondrial gene cytochrome oxidase subunit I (COI), the standard vertebrate barcoding gene, was amplified by polymerase chain reaction (PCR) using the primers dgLCO-1490 and dgHCO-2198 (Meyer, 2003). PCR products were visualized via 1.5% agarose gel electrophoresis. Unincorporated nucleotides were removed from the PCR product using 2 μL of ExoSAP-IT^®^ per sample, and sequencing (forward and reverse) was carried out via Eurofins SimpleSeq DNA Sequencing service (Louisville, KY, United States) following manufacturer’s protocols. Chromatograms were checked manually and sequences were assembled using Geneious v.7.1.7 (Kearse et al., 2012). Sequence alignment was carried out in MEGA7 (Kumar et al., 2016) using the ClustalW algorithm (Thompson et al., 1994) and sequences were manually checked to ensure there were no indels or stop codons. Two datasets were formed: a “full” dataset consisting of all sequences, and a “reduced” dataset consisting only of unique haplotypes. The numbers of conserved, variable, and parsimony-informative sites (not including the outgroup *Anolis allisoni*) were calculated in MEGA7.

### Phylogenetic Analyses

Sequence divergences were estimated using uncorrected p-distances and under the K2P model, a model extensively used in barcoding studies (e.g., Liu et al., 2015; Hawlitscheck et al., 2016), with 1000 bootstrap estimates in MEGA7. Recent publications have discussed the widespread misuse of the K2P model in barcoding analyses and its tendency to underestimate diversity (e.g., Barley and Thomson, 2016; Zinger and Philippe, 2016). Therefore, we selected the best-fit model of nucleotide substitution for the dataset using jModelTest 2.0 (Darriba et al., 2012) based on the Bayesian Information Criterion. TrN+G was selected (Tamura and Nei, 1993; Δ = 4.65) as the best-fit model (TIM1+I+G) was not implementable in MEGA7. We then estimated sequence divergences a third time using this model. Between group and within group averages were taken from initial *a priori* identifications and by categorizing sequences to lineages as assigned by ABGD at a 7.2% threshold and the mPTP analysis (see below). These three groupings were chosen as conservative exemplars of *a priori*, distance-based, and tree-based delimitation methods, together with the fact that these produced congruent results between the “full” and “reduced” datasets (see below). We generated histograms of pairwise distances for all three models in R v3.2.3 (R Core Team, 2015) using the “*checkDNAbcd”* function in the package *ad hoc* (Sonet et al., 2013). We tested for possible substitution saturation and plotted transitions and transversions against K2P distances (Kimura, 1980) using DAMBE v.6.3.3 (Xia and Lemey, 2009; Xia, 2013). The first and second codon positions, the third codon position, and all codon positions were tested separately.

Evolutionary model-based hypotheses of phylogenetic relationships were estimated for these data. The goal of these analyses were not to infer evolutionary histories of the taxa using only single-locus datasets, but to provide a framework in which to test species delimitation methods. Both the reduced and full dataset were subjected to maximum likelihood (ML) phylogenetic analyses using the program RAxML v7.2.8 (Stamatakis, 2014), performed with 1,000 bootstrap replicates under the GTR+GAMMA substitution model with data partitioned by codon position. Samples of *Anolis allisoni* were included as the outgroup. To create an ultrametric gene tree for GMYC analyses (see below), we analyzed both datasets in BEAST v.2.4.7 (Bouckaert et al., 2014), using a strict clock, yule tree prior, a GTR+GAMMA model of substitution, and all other priors left at default values. Analyses were performed for 100 million generations, sampling every 5000. Tracer v.1.6 (Rambaut et al., 2014) was used to assess convergence and adequate posterior sampling (ESS > 200), and a maximum clade credibility tree was created using TreeAnnotator v.2.4.7 (Bouckaert et al., 2014) using mean heights for annotation.

### Species Delimitation Analyses

We tested three tree-based methods of species delimitation on both the full and reduced datasets: bPTP (Zhang et al., 2013), multi-rate PTP (mPTP; Kapli et al., 2017), and the single threshold general mixed Yule coalescent model (GYMC; Pons et al., 2006; Fujisawa and Barraclough, 2013). bPTP analyses were performed on the online server^1^ using the ML trees from RAxML. We ran 500,000 generations with a thinning of 500 and a burn-in of 0.1, then assessed convergence visually using the MCMC iteration v. log-likelihood plots generated automatically. Next, we applied the recently introduced mPTP method, which improves upon the Poisson Tree Processes (PTP; Zhang et al., 2013) for single-locus species delimitation, to our datasets. Instead of all species sharing the same rate of evolution (λ) as in PTP, each species branch has its own λ in the mPTP model. This method determines which number of species fits best to the given data by utilizing the Akaike Information Criterion (rather than a *p*-value test as in PTP) because of the different number of parameters. mPTP has been shown to be consistent and very effective for species delimitation in datasets with uneven sampling (Blair and Bryson, 2017). Using the ML trees from RAxML, we performed four simultaneous Markov Chain Monte Carlo (MCMC) runs of 100,000,000 steps, sampling every 10,000 steps, to assess Average Support Values (ASV), the confidence of the ML delimitation for each species. Convergence of the runs was assessed visually using the outputted likelihood plot of the combined runs (created using the “-*-mcmc_log*” command) and the Average Standard Deviation of Delimitation Support Values (ASDDSV), which approaches zero as the multiple MCMC runs converge on the same delimitation distribution. Finally, we incorporated the single threshold GMYC model for our full and reduced datasets using the summarized BEAST ultrametric trees. These analyses were performed using the R package *‘splits’* (Ezard et al., 2009).

To compare tree-based and distance-based methods of species delimitation, as well as statistically detect the barcode gap in our data, we then performed two distance-based methods: the Refined Single Linkage algorithm (RESL; Ratnasingham and Hebert, 2013) and the Automated Barcode Gap Discovery method (ABGD; Puillandre et al., 2012). RESL was implemented directly in the Barcode of Life Datasystem (Ratnasingham and Hebert, 2007, 2013)^2^, and used to assign sequences to OTUs. ABGD infers a model-based confidence limit for intraspecific divergence based on prior intraspecific divergences, clustering similar haplotypes together as “species.” The barcode gap is the first significant gap beyond the intraspecific divergence limit, and therefore two samples taken from distinct clusters will have a distance between them larger than the barcoding gap. This inference and gap detection is then continuously applied to the previous clusters until a final partition is reached (Guarnizo et al., 2015). Our alignments were processed in ABGD web^3^ using the Kimura two-parameter substitution model, prior for maximum value of intraspecific divergence between 0.001 and 0.1, 15 recursive steps, and a gap width (X) of 1.0.

Following Ahrens et al. (2016) and Blair and Bryson (2017), we calculated the match ratio for each analyses as follows:

$$match\;ratio=2\times \frac{N_{match}}{\left(N_{delimited}+N_{morph}\right)}$$

In this formula, *N_match_* refers to the number of delimited species that exactly match a taxonomically defined morphospecies (not including taxa split as cryptic lineages), *N_delimited_* refers to the total number of lineages delimited by an analysis, and *N_morph_* refers to the total number of morphologically defined species (morphospecies). The use of a match ratio provides a better comparable value for different analyses than simply reporting the *N_match_*, where splitting and lumping species cancels out match values.

## Results

We successfully amplified 410 samples representing 33 native species of *Norops* and two samples of *Anolis allisoni* for COI, generating a full dataset of 412 sequences. Twenty-nine of the 34 total nominal species were represented by two or more samples, with five species represented by a single sample. Sequences ranged in length from 521 to 654 bp, with 395 sequences (95.9%) longer than 600 bp. Three hundred fifty-two sites (53.8%) were conserved, 302 (46.2%) were variable, and 281 (43.0%) were parsimony-informative. After removing redundant identical haplotypes, the reduced dataset consisted of 290 unique sequences (288 ingroup, 2 outgroup).

For tests of substitution saturation on all codon positions and codon position 1 and 2, index of substitution saturation (Iss) values were less than Issc.Sym (critical index of substitution saturation assuming a symmetrical topology) or Issc.Asym (critical index of substitution saturation assuming an asymmetrical topology) for all numbers of species simulated (NumOTU), suggesting little substitution saturation has occurred (Supplementary Table 2 and Supplementary Figure 1). Tests on only codon position 3, however, resulted in Iss values higher than Issc.Asym values for NumOTU values of 16 and 32. This suggests there potentially is saturation of the 3rd codon position for the dataset. DAMBE is limited to NumOTU ≤ 32, but tests random subsets of 4, 8, 16, and 32 OTUs multiple times in order to circumvent this limitation (Xia and Lemey, 2009).

**FIGURE 2:**
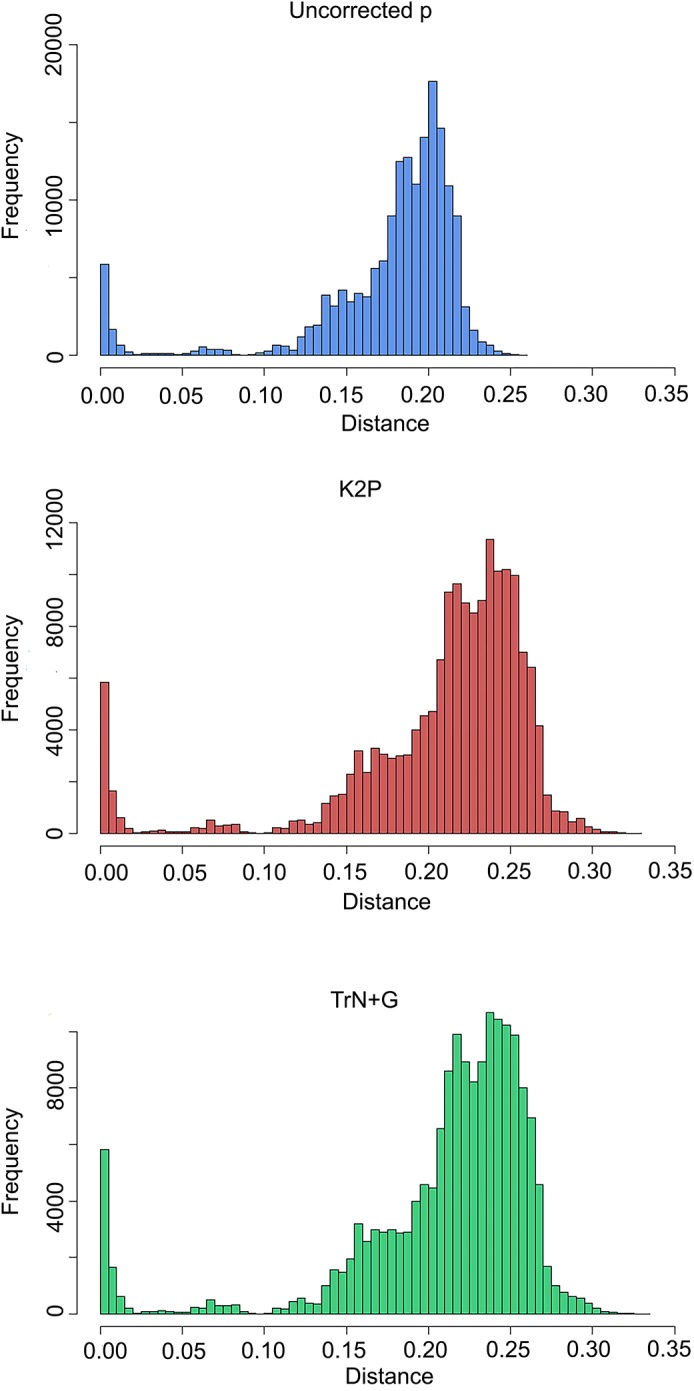
Histograms of pairwise distances under different models of nucleotide substitution: uncorrected **(top)**, K2P **(middle)**, and TrN+G **(bottom)**.

Summaries of uncorrected pairwise distances, K2P-corrected distances, and TrN+G corrected distances are shown in Table 2 and Figure 2, with the full data available in Supplementary Data Sheet 1. Generating pairwise distances using the better-fit model (TrN+G) resulted in larger values than both uncorrected pairwise and K2P models, increasing the mean distance between samples from 0.171 (uncorrected p) to 0.211. Assigning sequences to lineages recovered by ABGD at a 7.2% threshold tightened the average intra- and interspecific distances, greatly improving the delineation of a barcoding gap threshold regardless of the model implemented. Assignment of samples by mPTP similarly tightened the range of average intraspecific distances, but led to a wider range of interspecific distances.

**Table 2:** Summaries of uncorrected pairwise distances, K2P-corrected distances, and TrN+G-corrected distances for the dataset, including averages based on *a priori* identification (morphospecies), ABGD clustering (at 7.2% threshold), and mPTP delimitations.

	Whole dataset			Averages within groups (Intraspecific)			Averages between groups (Interspecific)		
			
Model used	Range	Mean	StDev	Range	Mean	StDev	Range	Mean	StDev
**Uncorrected *p***	0.0–0.247	0.171	0.047						
*a priori*				0.0–0.062	0.014	0.016	0.049–0.242	0.181	0.024
ABGD				0.0–0.037	0.011	0.010	0.066–0.242	0.181	0.025
mPTP				0.0–0.030	0.006	0.006	0.031–0.242	0.180	0.028
**K2P**	0.0–0.313	0.200	0.058						
*a priori*				0.0–0.068	0.015	0.017	0.051–0.300	0.212	0.032
ABGD				0.0–0.039	0.011	0.011	0.070–0.301	0.212	0.033
mPTP				0.0–0.032	0.006	0.006	0.032–0.305	0.211	0.037
**TrN+G**	0.0–0.336	0.211	0.062						
*a priori*				0.0–0.070	0.015	0.018	0.052–0.323	0.224	0.035
ABGD				0.0–0.039	0.011	0.011	0.071–0.323	0.223	0.036
mPTP				0.0–0.033	0.006	0.007	0.032–0.326	0.222	0.040


**FIGURE 3:**
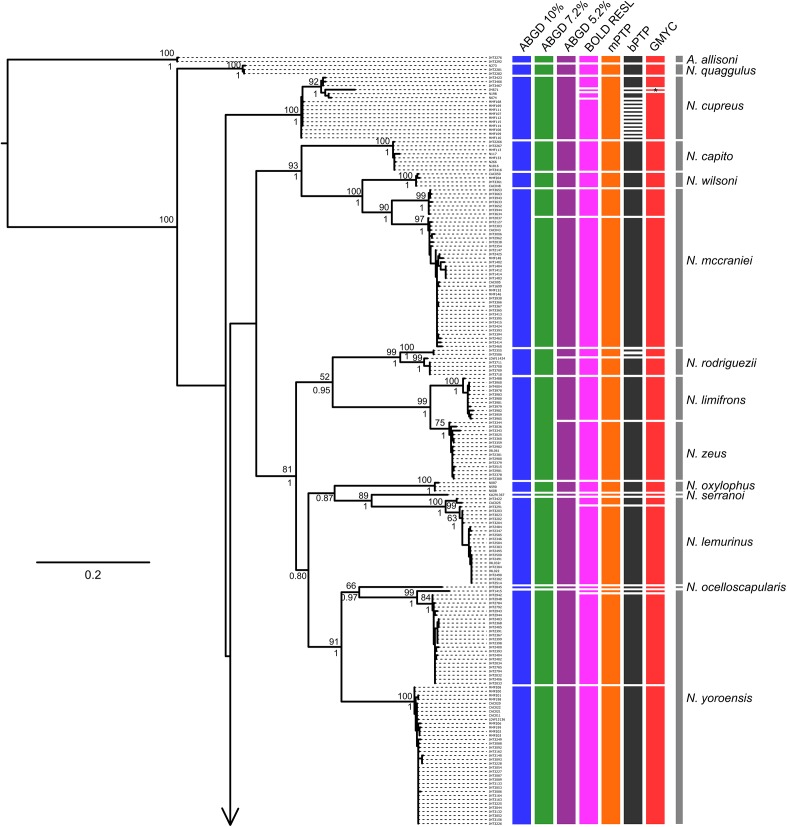
Maximum likelihood phylogeny of the full COI sequence dataset, with lineage assignments from the three tree-based (mPTP, bPTP, GMYC) and two distance-based (RESL, ABGD at three thresholds) methods. ML bootstrap support and Bayesian posterior probabilities shown when ≥ 50 and 0.50, respectively. Gray bars span all samples assigned *a priori* to named taxa (morphospecies). Note (^∗^): JMS71 was assigned to its own lineage by GMYC, apart from all other samples assigned to *N. cupreus* (i.e., there were two inferred lineages within *N. cupreus*, not three).

**FIGURE 4:**
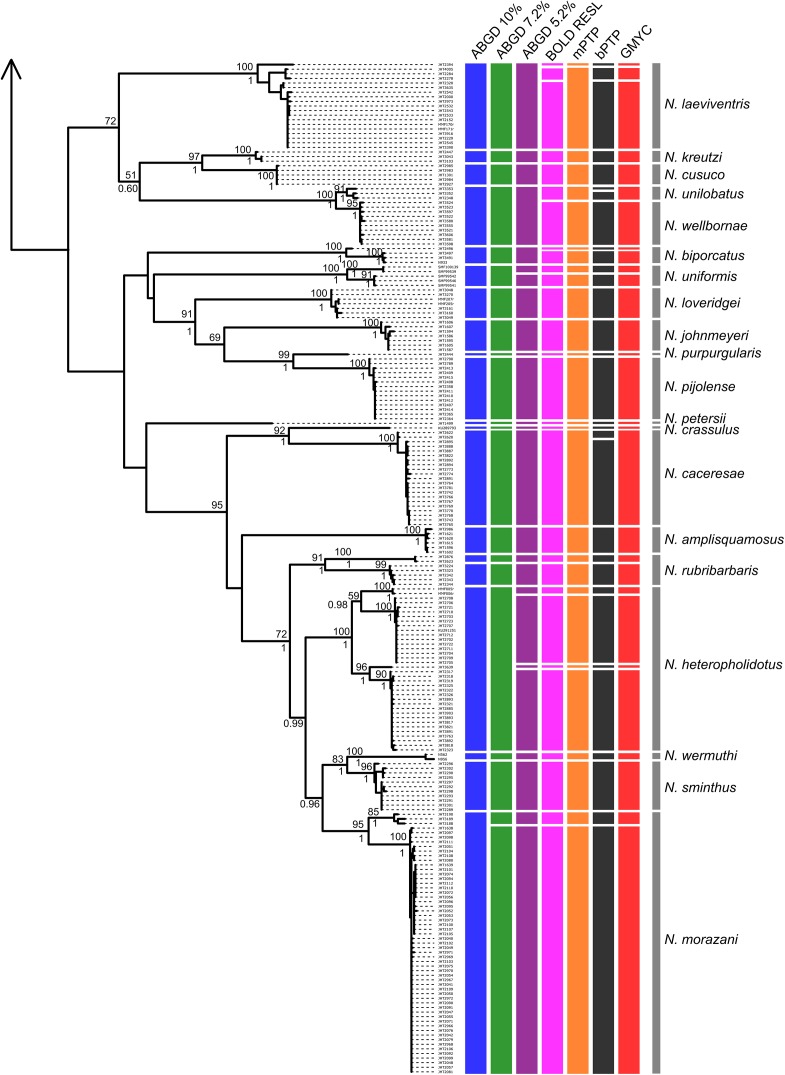
Maximum likelihood phylogeny of the full COI sequence dataset, with lineage assignments from the three tree-based (mPTP, bPTP, GMYC) and two distance-based (RESL, ABGD at three thresholds) methods (*continued from Figure 3*). ML bootstrap support and Bayesian posterior probabilities shown when ≥ 50 and 0.50, respectively. Gray bars span all samples assigned *a priori* to named taxa (morphospecies).

Tree- and distance-based methods of species delimitation did not produce congruent results, often inferring different numbers of species with the same method applied to the full and reduced datasets (Table 3 and Supplementary Tables 3, 4). Methods applied to the reduced dataset (290 sequences) inferred between 36 (ABGD threshold of 7.2%) and 59 (bPTP) species, while the same methods applied to the full dataset (412 sequences) inferred between 34 (ABGD threshold of 10%) and 64 (bPTP) species (Table 3 and Figure 3, 4). Match ratios of analyses performed on the reduced dataset were higher than their complement performed on the full dataset in all cases except the ABGD threshold of 7.2% (higher in full) and RESL (equal). Across both datasets, ABGD match ratios were the highest of all analyses, while mPTP match ratios were highest among tree-based analyses. Results of ABGD at 7.2% (36 inferred species), RESL (51), and mPTP (46; confidence interval 44–49) were congruent across both datasets. GMYC inferred 50 (full dataset; confidence interval 50–55; match ratio 0.548) and 51 (reduced dataset; confidence interval 50–54; match ratio 0.565) species. bPTP recovered an unreasonably high number of species with wide confidence intervals in both the full (64: 53–80) and reduced (59: 53–78) datasets, and had the lowest match ratios of any analysis (full: 0.429; reduced: 0.452). Several previous studies have recommended the use of 10% threshold when interpreting ABGD results (Kekkonen and Hebert, 2014; Blair and Bryson, 2017). This threshold inferred 34 nominal species and the highest match ratio (0.824) when applied to our full dataset, failing to delimit *N. unilobatus* from *N. wellborane* or *N. limifrons* from *N. zeus*, but delimiting two OTUs within both *N. rubribarbaris* and *N. yoroensis* (Figure 3, 4).

**Table 3:** Number of putative species (OTUs) inferred by each delimitation method from input datasets (“Full”: 412 sequences; “Reduced”: 290 unique sequences) of 34 nominal taxa, with exact matches (*N_match_*) and match ratios.

	*Distance-based*				*Tree-based*		
		
	ABGD 10.0%	ABGD 7.2%	ABGD 5.2%	BOLD RESL	mPTP	bPTP	GMYC
**Full Dataset**	**34**	**36**	**42**	**51**	**46** (44–49)	**64** (53–80)	**50** (50–55)
*N_match_*	28	26	25	23	24	21	23
*Match ratio*	0.824	0.743	0.658	0.541	0.600	0.429	0.548
**Reduced Dataset**	**–**	**36**	**38**	**51**	**46** (44–49)	**59** (53–78)	**51** (50–54)
*N_match_*	–	25	24	23	25	21	24
*Match ratio*	–	0.714	0.667	0.541	0.625	0.452	0.565


*Confidence intervals for the number of clusters delimited are provided when available. Please note that ABGD at a 10.0% threshold returned no results (0 groups) for the “reduced” dataset (see Supplementary Figures 2, 3).*

## Discussion

### Species Delimitation Method Performance

Numerous studies have compared and contrasted the single-locus delimitation methods tested here across empirical and simulated datasets (e.g., Talavera et al., 2013; Dellicour and Flot, 2015, 2018; Ahrens et al., 2016; Blair and Bryson, 2017; Luo et al., 2018). Our results are consistent with many empirical studies showing that different methods often produce different delimitation scenarios when using single-locus data. Widely used methods of single-locus species delimitation tested here are each subject to the potential biases and differing conditions inherent in empirical datasets.

Despite recovering largely incongruent numbers of OTUs, the methods were consistent in recovering more OTUs than the number of species originally considered (Figure 3, 4 and Table 3). Given the rapid evolution of mainland anoles and the lack of clarity regarding the relationships of all populations analyzed, it is not surprising that more lineages were inferred than are currently recognized. While some of these OTUs might correspond to undescribed cryptic species, it is also likely that some structure is a result of genetic drift or isolated populations currently undergoing speciation. In some cases, however, it is clear that the analyses over-split taxa. As in Blair and Bryson (2017)’s analyses of *Phrynosoma* lizards, our bPTP analyses produced unreasonable delimitations with wide confidence intervals; some of these clusters do not reflect relationships as understood with better molecular sampling (Hofmann and Townsend, 2017) and others were separated into numerous lineages despite little-to-no divergence between them (e.g., *N. cupreus;* Figure 3). bPTP is known to be sensitive to different mutation rates, but unlike in the simulated data of Dellicour and Flot (2018), here it produced the least-accurate delimitations.

Several factors might have influenced the incongruent results among the other algorithms. ABGD is known to overlump, performing poorly on more speciose datasets with faster speciation rates compared to smaller, more slowly speciating populations (Dellicour and Flot, 2015, 2018). ABGD’s conservative tendencies have also been shown across a variety of loci and taxa, including amphibians (Guarnizo et al., 2015), other lizards (Blair and Bryson, 2017), brittle stars (Boissin et al., 2017), and insects (e.g., Song et al., 2018). Here, ABGD recovered the fewest inferred species and had the highest match ratios at all levels, but in no instance was the delimitation completely congruent with current taxonomy.

In contrast to ABGD’s overlumping, single-threshold GMYC is known to oversplit species (Talavera et al., 2013), as higher substitution rates, uneven sampling (including singletons or the inclusion of identical sequences), variation in population size among species, ongoing gene flow, or unresolved nodes could bias results (Esselstyn et al., 2012; Tang et al., 2014; Ahrens et al., 2016; Blair and Bryson, 2017; Luo et al., 2018). Five of the species sampled herein were represented by only a single sequence; in combination with variable effective sample sizes of these anoles, these samples might have contributed to the more liberal GMYC delimitations (Ahrens et al., 2016). This method recovered more clusters than distance-based analyses across our data, as well as in studies of Socotran reptiles (Vasconcelos et al., 2016), *Madascincus* lizards (Miralles and Vences, 2013), and *Ophiomorus* geckos (Korniolios et al., 2018). Interestingly, Blair and Bryson (2017) found it to be the most conservative method applied to their data.

The use of multiple models of nucleotide substitution (uncorrected pairwise, K2P, and TrN+G) resulted in a wide range of observed distances within the samples, with TrN+G estimating the widest range of distances. A difference of greater than 9% at the upper limit of interspecific differences is substantial enough to suggest the incorporation of multiple models as a standard practice in barcoding investigations, including those traditionally used models (uncorrected p and K2P), as well as the best fit model, as suggested by several authors (Srivathsan and Meier, 2012; Barley and Thomson, 2016; Zinger and Philippe, 2016). Regardless of the model of nucleotide substitution used, a clear barcoding gap was recovered by assigning samples based on delimitation methods rather than *a priori* (Table 2). The use of either ABGD or mPTP delimitations improved distance analyses, tightening intraspecific average distances across the board. A proposed COI barcoding gap between 3.7 and 7.1% could be considered for these anoles, though we caution against a strict cutoff as it is clear further taxonomic investigation is necessary in order to more clearly delineate a barcoding gap in these taxa.

The incongruous results of different methods in many empirical studies emphasizes the importance of using multiple species delimitation methods for single-locus data; using one line of evidence might serve only to increase the confusion regarding species boundaries. While one method might be advantageous due to performance or speed, we agree with Dellicour and Flot (2018) that one should compare the results of several approaches when attempting to draw conclusions from single-locus data. Similarly, researchers should carefully consider the models of nucleotide substitution used, as well as the effect of reducing their datasets down to unique haplotypes has on their analyses and inferences. Here, we demonstrate that the same method applied to a full dataset can return different results compared to one consisting only of non-redundant haplotypes, in some cases overlumping or oversplitting lineages.

### Taxonomic Implications

Analyses of a single mitochondrial locus should not be the sole line of evidence used to draw taxonomic conclusions (DeSalle, 2006), just as using any single character to dictate systematic changes is ill-advised (Will et al., 2005). Analyzing a single gene barcode as a first step in identifying previously overlooked lineages, however, complements taxonomic investigation, providing a roadmap for groups of taxa in need of more complete study. Furthermore, barcoding analyses can be useful in identifying areas of population structure and potential areas where gene flow is not occurring between populations (Tavares and Baker, 2008). In poorly represented taxa, a single gene can test the efficacy of groupings based solely on morphology.

Across all analyses, no method inferred species limits completely congruent with current taxonomy, even under the most conservative considerations. Based on the performance of the multiple methods on both datasets, we consider ABGD at a 7.2% threshold to be the most reasonable distance-based estimate and mPTP to be the most reasonable tree-based methods of delimitation for this dataset; these methods inferred 36 and 46 lineages, respectively, and were congruent between full and reduced datasets. These estimates suggest that species diversity in Chortís Block anoles could be underestimated by as much as 26%. The recently described *N. caceresae* (Hofmann and Townsend, 2018) was supported by every analysis. Several recognized species were inferred by these methods to represent two or more OTUs, each of which appear to represent reciprocally monophyletic populations often separated biogeographically. These methods congruently inferred multiple geographically isolated species level lineages within *Norops mccraniei, N. morazani, N. rubribarbaris*, and *N. yoroensis.* mPTP additionally inferred multiple lineages within *N. biporcatus, N. heteropholidotus, N. laeviventris, N. lemurinus, N. rodriguezii*, and *N. uniformis.* Conversely, ABGD at a 7.2% threshold inferred *N. limifrons* and *N. zeus* as the same lineage, but as distinct lineages at a 5.2% threshold; *N. unilobatus* and *N. wellbornae* were similarly considered a single lineage by this method. These results suggest that further taxonomic investigation into these lineages, including more thorough molecular sampling, is warranted.

## Conclusion

In this study, we utilized a COI-barcode dataset of mainland anoles to test inferences of several commonly utilized single-locus delimitation methods. Our results provide further support for the necessity of utilizing multiple methods in barcoding studies, as results differed between tree- and distance-based analyses, as well as between the same analyses applied to a full dataset and one with the redundant haplotypes removed. Additionally, we highlight previously unrecognized diversity in need of further taxonomic and systematic investigation. Finally, a goal of this study was to provide a foundation for further studies of Chortís Block anoles by providing a reference library of barcodes to allow for the efficient identification of difficult-to-assign specimens, particularly females and juveniles. While a tremendous amount of work remains to be done if our understanding of mainland anoles will ever rival that of Caribbean anoles, the stage is set for a generation of researchers to undertake that effort.

## Data Availability Statement

The sequence data generated for this study has been deposited in GenBank. See Supplementary Table 1 for accession numbers.

## Ethics Statement

This study was carried out in accordance with the recommendations of Indiana University of Pennsylvania Institutional Animal Use and Care Committee (IUP-IACUC) and the University of Florida IFAS Animal Research Committee (UF-ARC). Protocols were approved by the IUP-IACUC and UF-ARC.

## Author Contributions

EH and JT designed the study. All authors collected and contributed samples. EH, IL-M, MM-F, and JT generated the molecular data. EH analyzed the data. EH and JT wrote the first draft of the manuscript. All authors reviewed, edited, and contributed to the final manuscript.

## Conflict of Interest Statement

The authors declare that the research was conducted in the absence of any commercial or financial relationships that could be construed as a potential conflict of interest.

## Footnotes

1 http://species.h-its.org/

2 http://www.boldsystems.org/

3 http://wwwabi.snv.jussieu.fr/public/abgd/abgdweb.html
