# Supplementary material for: Cryptic Diversity, but to What Extent? Discordance Between Single-Locus Species Delimitation Methods Within Mainland Anoles (Squamata: Dactyloidae) of Northern Central America
Source: Front Genet. 2019 Feb 11;10:11. doi: 10.3389/fgene.2019.00011 (PMC6378269; doi:10.3389/fgene.2019.00011)
Supplement: Supplementary file 1 [file Data_Sheet_1.pdf]

Supplementary Material

Cryptic diversity, but to what extent? Discordance between single-locus species delimitation methods within mainland anoles (Squamata: Dactyloidae) of Northern Central America

Erich P. Hofmann, Kirsten E. Nicholson, Ileana R. Luque-Montes, Gunther Köhler, César A. Cerrato-Mendoza, Melissa Medina-Flores, Larry David Wilson, Josiah H. Townsend*

*** Correspondence:** Josiah H. Townsend: josiah.townsend@iup.edu

1 Supplementary Tables and Figures

1.1 Supplementary Tables

Supplementary Table 1. Full sampling data for the COI Barcoding analysis, with Museum and GenBank accession numbers**.**

SPECIES	FIELD #	MUSEUM #	COUNTRY	DEPARTMENT	GENBANK
Anolis allisoni	JHT3276	USNM 578739	Honduras	Islas de la Bahía	KU687938
Anolis allisoni	JHT3292	USNM 578740	Honduras	Islas de la Bahía	KU687939
Norops amplisquamosus	JHT1596	UF 149642	Honduras	Cortés	KU687941
Norops amplisquamosus	JHT1602	UF 149643	Honduras	Cortés	MH449707
Norops amplisquamosus	JHT1615	UF 149645	Honduras	Cortés	KU687942
Norops amplisquamosus	JHT1620	UF 149647	Honduras	Cortés	KU687940
Norops amplisquamosus	JHT1621	UF 149648	Honduras	Cortés	KU687943
Norops amplisquamosus	JHT2986	USNM 578741	Honduras	Cortés	KU687944
Norops biporcatus	JHT2496	-	Honduras	Cortés	KU687946
Norops biporcatus	JHT3491	-	Honduras	Olancho	KU687947
Norops biporcatus	JHT3497	-	Honduras	Olancho	KU687945
Norops biporcatus	N933	UF 156021	Nicaragua	Jinotega	MH449971
Norops capito	JHT2266	UF 156378	Nicaragua	Matagalpa	MH449756
Norops capito	JHT2267	UF 156379	Nicaragua	Matagalpa	MH449757
Norops capito	JHT3416	CM 161327	Honduras	Olancho	MH449879
Norops capito	MMF113	-	Honduras	Olancho	MH449943
Norops capito	MMF133	CM 161377	Honduras	Olancho	MH449947
Norops capito	N1016	UF 156024	Nicaragua	Jinotega	MH449972

Norops capito	N117	UF 156301	Nicaragua	R.A.A. Norte	MH449964
Norops capito	N266	UF 156302	Nicaragua	Jinotega	MH449966
Norops crassulus	-	KU 289793	El Salvador	Santa Ana	MF094524
Norops caceresae	JHT2620	UF 166186	Honduras	La Paz	KU687954
Norops caceresae	JHT2622	UF 166188	Honduras	La Paz	KU687955
Norops caceresae	JHT2773	UF 166190	Honduras	Intibucá	KU687949
Norops caceresae	JHT2774	UF 166191	Honduras	Intibucá	KU687950
Norops caceresae	JHT2891	-	Honduras	Intibucá	KU687951
Norops caceresae	JHT2892	-	Honduras	Intibucá	KU687952
Norops caceresae	JHT2894	-	Honduras	Intibucá	MF094513
Norops caceresae	JHT2895	-	Honduras	Intibucá	KU687956
Norops caceresae	JHT3742	CM 161304	Honduras	Intibucá	MH449905
Norops caceresae	JHT3743	CM 161305	Honduras	Intibucá	MH449906
Norops caceresae	JHT3764	MVZ:Herp:286121	Honduras	Intibucá	MH449908
Norops caceresae	JHT3765	-	Honduras	Intibucá	MH449909
Norops caceresae	JHT3766	CM 161306	Honduras	Intibucá	MH449910
Norops caceresae	JHT3767	MVZ:Herp:286122	Honduras	Intibucá	MH449911
Norops caceresae	JHT3768	MVZ:Herp:286123	Honduras	Intibucá	MF094598
Norops caceresae	JHT3769	MVZ:Herp:286124	Honduras	Intibucá	MH449912
Norops caceresae	JHT3770	CM 161307	Honduras	Intibucá	MF094599
Norops caceresae	JHT3781	CM 161308	Honduras	Intibucá	MH449913
Norops caceresae	JHT3822	CM 161309	Honduras	Intibucá	MH449916
Norops caceresae	JHT3887	CM 161310	Honduras	Intibucá	MH449917
Norops caceresae	JHT3888	CM 161311	Honduras	Intibucá	MF094519
Norops cupreus	JHT3423	CM 161329	Honduras	Colon	MH449881
Norops cupreus	JHT3466	CM 161330	Honduras	Colon	MH449882
Norops cupreus	JHT3467	CM 161331	Honduras	Colon	MH449883
Norops cupreus	JMS71	-	Costa Rica	Guanacaste	MH449933
Norops cupreus	MMF107	-	Honduras	Olancho	MH449937
Norops cupreus	MMF108	-	Honduras	Olancho	MH449938
Norops cupreus	MMF109	-	Honduras	Olancho	MH449939
Norops cupreus	MMF110	-	Honduras	Olancho	MH449940
Norops cupreus	MMF111	-	Honduras	Olancho	MH449941
Norops cupreus	MMF112	-	Honduras	Olancho	MH449942
Norops cupreus	MMF114	-	Honduras	Olancho	MH449944
Norops cupreus	MMF115	-	Honduras	Olancho	MH449945
Norops cupreus	MMF168	CM 161378	Honduras	Olancho	MH449950
Norops cupreus	MMF169	CM 161379	Honduras	Olancho	MH449951

Norops cupreus	N198	UF 166862	Nicaragua	R.A.A. Norte	MH449965
Norops cupreus	N674	UF 156063	Nicaragua	Jinotega	MH449970
Norops cusuco	JHT1301	UF 142740	Honduras	Cortés	KU687958
Norops cusuco	JHT2927	UF 166193	Honduras	Cortés	KU687959
Norops cusuco	JHT2983	USNM 578743	Honduras	Cortés	KU687961
Norops cusuco	JHT2984	USNM 578744	Honduras	Cortés	KU687957
Norops cusuco	JHT2985	USNM 578745	Honduras	Cortés	KU687960
Norops heteropholidotus	MMF005	SMF 103928	Honduras	Ocotepeque	MF094527
Norops heteropholidotus	MMF006	SMF 103929	Honduras	Ocotepeque	KU687966
Norops heteropholidotus	JHT2704	UF 166196	Honduras	Ocotepeque	KU687978
Norops heteropholidotus	JHT2705	UF 166197	Honduras	Ocotepeque	MF094508
Norops heteropholidotus	JHT2706	UF 166198	Honduras	Ocotepeque	KU687963
Norops heteropholidotus	JHT2707	UF 166199	Honduras	Ocotepeque	KU687972
Norops heteropholidotus	JHT2708	UF 166200	Honduras	Ocotepeque	MF094509
Norops heteropholidotus	JHT2709	UF 166201	Honduras	Ocotepeque	KU687965
Norops heteropholidotus	JHT2710	UF 166202	Honduras	Ocotepeque	MF094510
Norops heteropholidotus	JHT2711	UF 166203	Honduras	Ocotepeque	MF094511
Norops heteropholidotus	JHT2712	UF 166204	Honduras	Ocotepeque	KU687964
Norops heteropholidotus	JHT2721	UF 166207	Honduras	Ocotepeque	KU687974
Norops heteropholidotus	JHT2722	UF 166208	Honduras	Ocotepeque	MF094512
Norops heteropholidotus	JHT2723	UF 166209	Honduras	Ocotepeque	KU687975
Norops heteropholidotus	-	KU 291251	El Salvador	Chalatenango	MF094526
Norops heteropholidotus	JHT2702	UF 166194	Honduras	Ocotepeque	KU687970
Norops heteropholidotus	JHT2703	UF 166195	Honduras	Ocotepeque	KU687973
Norops heteropholidotus	JHT2317	UF 166279	Honduras	Intibucá	KU687962
Norops heteropholidotus	JHT2319	UF 166281	Honduras	Intibucá	MF094505
Norops heteropholidotus	JHT2321	UF 166283	Honduras	Intibucá	KU687971
Norops heteropholidotus	JHT2322	UF 166284	Honduras	Intibucá	KU687976
Norops heteropholidotus	JHT2323	UF 166285	Honduras	Intibucá	KU687977
Norops heteropholidotus	JHT2325	UF 166287	Honduras	Intibucá	MF094506
Norops heteropholidotus	JHT2326	UF 166288	Honduras	Intibucá	MF094507
Norops heteropholidotus	JHT2885	-	Honduras	Intibucá	KU687969
Norops heteropholidotus	JHT2893	-	Honduras	Intibucá	KU687967
Norops heteropholidotus	JHT3763	CM 161349	Honduras	Intibucá	MH449907
Norops heteropholidotus	JHT3817	CM 161350	Honduras	Intibucá	MH449914
Norops heteropholidotus	JHT3818	CM 161351	Honduras	Intibucá	MF094517
Norops heteropholidotus	JHT3821	CM 161352	Honduras	Intibucá	MH449915
Norops heteropholidotus	JHT3891	CM 161353	Honduras	Intibucá	MF094520

Norops heteropholidotus	JHT3892	CM 161354	Honduras	Intibucá	MF094521
Norops heteropholidotus	JHT3893	CM 161355	Honduras	Intibucá	MF094522
Norops heteropholidotus	JHT3903	CM 161365	Honduras	Intibucá	MF094523
Norops heteropholidotus	JHT2318	UF 166280	Honduras	Intibucá	KU687968
Norops heteropholidotus	JHT3639	CM 161344	Honduras	Lempira	MF094514
Norops johnmeyeri	JHT1586	UF 149554	Honduras	Cortés	KU687981
Norops johnmeyeri	JHT1587	UF 149555	Honduras	Cortés	MH449705
Norops johnmeyeri	JHT1594	UF 149556	Honduras	Cortés	KU687980
Norops johnmeyeri	JHT1595	UF 149557	Honduras	Cortés	MH449706
Norops johnmeyeri	JHT1605	UF 149559	Honduras	Cortés	KU687982
Norops johnmeyeri	JHT1606	UF 149560	Honduras	Cortés	KU687983
Norops johnmeyeri	JHT1607	UF 149561	Honduras	Cortés	KU687979
Norops kreutzi	JHT2447	UF 166212	Honduras	Yoro	KU687984
Norops kreutzi	JHT3043	USNM 578819	Honduras	Atlántida	KU687985
Norops kreutzi	JHT3103	USNM 578749	Honduras	Atlántida	MH449845
Norops laeviventris	JHT2000	UF 150154	Honduras	Francisco Morazán	KU687998
Norops laeviventris	JHT2152	-	Honduras	Francisco Morazán	KU687995
Norops laeviventris	JHT2229	-	Honduras	Francisco Morazán	KU687996
Norops laeviventris	JHT2278	-	Nicaragua	Matagalpa	KU687986
Norops laeviventris	JHT2284	-	Nicaragua	Matagalpa	KU687992
Norops laeviventris	JHT2320	UF 166282	Honduras	Intibucá	MH449762
Norops laeviventris	JHT2532	UF 166289	Honduras	Comayagua	KU687993
Norops laeviventris	JHT2533	UF 166290	Honduras	Comayagua	MH449806
Norops laeviventris	JHT2542	UF 166291	Honduras	Comayagua	KU687991
Norops laeviventris	JHT2543	UF 166292	Honduras	Comayagua	KU687997
Norops laeviventris	JHT2545	UF 166294	Honduras	Comayagua	KU687989
Norops laeviventris	JHT2973	UF 166214	Honduras	Francisco Morazán	KU687990
Norops laeviventris	JHT3390	CM 161325	Honduras	Olancho	KU687987
Norops laeviventris	JHT3635	CM 161343	Honduras	Lempira	MH449897
Norops laeviventris	JHT3916	CM 161374	Honduras	Francisco Morazán	MH449918
Norops laeviventris	JHT4005	CM 161376	Nicaragua	Jinotega	MH449932
Norops laeviventris	MMF170	CM 161380	Honduras	Olancho	KU687994
Norops laeviventris	MMF171	CM 161381	Honduras	Olancho	MH449952
Norops laeviventris	JHT2394	-	Honduras	Yoro	KU687988
Norops lemurinus	CAC025	USNM 578816	Honduras	Atlántida	MH449692
Norops lemurinus	JHT3422	CM 161328	Honduras	Colon	MH449880
Norops lemurinus	IRL022	UF 166215	Honduras	Cortés	MH449696
Norops lemurinus	IRL032	UF 166216	Honduras	Cortés	MH449697

Norops lemurinus	JHT2346	UF 166220	Honduras	Santa Bárbara	MH449763
Norops lemurinus	JHT2347	UF 166221	Honduras	Santa Bárbara	MH449764
Norops lemurinus	JHT2382	UF 166222	Honduras	Cortés	MH449776
Norops lemurinus	JHT2383	UF 166223	Honduras	Cortés	MH449777
Norops lemurinus	JHT2384	UF 166224	Honduras	Cortés	MH449778
Norops lemurinus	JHT2484	UF 166225	Honduras	Santa Bárbara	MH449799
Norops lemurinus	JHT2490	UF 166226	Honduras	Cortés	MH449800
Norops lemurinus	JHT2491	UF 166227	Honduras	Cortés	MH449801
Norops lemurinus	JHT2495	UF 166228	Honduras	Cortés	MH449802
Norops lemurinus	JHT2500	UF 156628	Honduras	Cortés	MH449803
Norops lemurinus	JHT2514	UF 156629	Honduras	Cortés	MH449804
Norops lemurinus	JHT2584	UF 166229	Honduras	Santa Bárbara	MH449808
Norops lemurinus	JHT2585	UF 166230	Honduras	Santa Bárbara	MH449809
Norops lemurinus	JHT3023	USNM 578750	Honduras	Atlántida	MH449831
Norops lemurinus	JHT3202	USNM 578751	Honduras	Atlántida	MH449853
Norops lemurinus	JHT3203	USNM 578752	Honduras	Atlántida	MH449854
Norops lemurinus	JHT3204	USNM 578753	Honduras	Atlántida	MH449855
Norops lemurinus	JHT3291	USNM 578760	Honduras	Islas de la Bahia	MH449862
Norops limifrons	JHT3488	-	Honduras	Olancho	MH449884
Norops limifrons	JHT3959	UNAN-León	Nicaragua	Jinotega	MH449922
Norops limifrons	JHT3960	UNAN-León	Nicaragua	Jinotega	MH449923
Norops limifrons	JHT3965	UNAN-León	Nicaragua	Jinotega	MH449924
Norops limifrons	JHT3978	UNAN-León	Nicaragua	Jinotega	MH449925
Norops limifrons	JHT3979	UNAN-León	Nicaragua	Jinotega	MH449926
Norops limifrons	JHT3980	UNAN-León	Nicaragua	Jinotega	MH449927
Norops limifrons	JHT3981	UNAN-León	Nicaragua	Jinotega	MH449928
Norops limifrons	JHT3982	UNAN-León	Nicaragua	Jinotega	MH449929
Norops limifrons	JHT3983	UNAN-León	Nicaragua	Jinotega	MH449930
Norops limifrons	JHT4004	UNAN-León	Nicaragua	Jinotega	MH449931
Norops loveridgei	JHT3048	USNM 578754	Honduras	Atlántida	MH449835
Norops loveridgei	JHT3049	USNM 578755	Honduras	Atlántida	MH449807
Norops loveridgei	JHT3160	USNM 578756	Honduras	Atlántida	KU688001
Norops loveridgei	JHT3161	USNM 578757	Honduras	Atlántida	KU687999
Norops loveridgei	JHT3270	USNM 578758	Honduras	Atlántida	KU688000
Norops loveridgei	MMF205	CM 158363	Honduras	Atlantida	KU688002
Norops loveridgei	MMF207	CM 158364	Honduras	Atlantida	MH449961
Norops mccraniei	CAC005	USNM 578770	Honduras	Francisco Morazán	MH449687
Norops mccraniei	CAC043	USNM 578772	Honduras	Comayagua	MH449693

Norops mccraniei	JHT1402	-	Honduras	Cortés	MH449699
Norops mccraniei	JHT1403	-	Honduras	Cortés	MH449700
Norops mccraniei	JHT1404	-	Honduras	Cortés	MH449701
Norops mccraniei	JHT1412	-	Honduras	Cortés	MH449702
Norops mccraniei	JHT1414	-	Honduras	Cortés	MH449703
Norops mccraniei	JHT1699	UF 176105	Honduras	Francisco Morazán	MH449710
Norops mccraniei	JHT2037	-	Honduras	Francisco Morazán	MH449714
Norops mccraniei	JHT2038	-	Honduras	Francisco Morazán	MH449715
Norops mccraniei	JHT2127	-	Honduras	Francisco Morazán	MH449754
Norops mccraniei	JHT2147	-	Honduras	Francisco Morazán	MH449755
Norops mccraniei	JHT2303	UF 166298	Honduras	Comayagua	MH449761
Norops mccraniei	JHT2354	-	Honduras	Santa Bárbara	MH449766
Norops mccraniei	JHT2424	UF 166299	Honduras	Yoro	MH449795
Norops mccraniei	JHT2425	UF 166300	Honduras	Yoro	MH449796
Norops mccraniei	JHT2460	UF 166303	Honduras	Yoro	MH449797
Norops mccraniei	JHT2462	UF 166305	Honduras	Yoro	MH449798
Norops mccraniei	JHT2962	UF 166308	Honduras	Francisco Morazán	MH449821
Norops mccraniei	JHT3006	USNM 578778	Honduras	Santa Bárbara	MH449830
Norops mccraniei	JHT3365	SMF 100104	Honduras	Olancho	MH449870
Norops mccraniei	JHT3366	SMF 100105	Honduras	Olancho	MH449871
Norops mccraniei	JHT3367	SMF 100106	Honduras	Olancho	MH449872
Norops mccraniei	JHT3393	SMF 100107	Honduras	Olancho	MH449873
Norops mccraniei	JHT3394	SMF 100108	Honduras	Olancho	MH449874
Norops mccraniei	JHT3395	SMF 100109	Honduras	Olancho	MH449875
Norops mccraniei	JHT3413	SMF 100110	Honduras	Olancho	MH449876
Norops mccraniei	JHT3414	SMF 100111	Honduras	Olancho	MH449877
Norops mccraniei	JHT3415	SMF 100112	Honduras	Olancho	MH449878
Norops mccraniei	JHT3930	CM 161375	Honduras	Francisco Morazán	MH449919
Norops mccraniei	MMF132	-	Honduras	Olancho	MH449946
Norops mccraniei	MMF146	SMF 100100	Honduras	Olancho	MH449948
Norops mccraniei	MMF148	SMF 100101	Honduras	Olancho	MH449949
Norops mccraniei	JHT3633	SMF 103885	Honduras	Lempira	MH449895
Norops mccraniei	JHT3634	SMF 103886	Honduras	Lempira	MH449896
Norops mccraniei	JHT3652	SMF 103887	Honduras	Lempira	MH449898
Norops mccraniei	JHT3653	SMF 103888	Honduras	Lempira	MH449899
Norops mccraniei	JHT3663	SMF 103889	Honduras	Lempira	MH449900
Norops mccraniei	JHT3943	SMF 103890	Honduras	Lempira	MH449920
Norops mccraniei	JHT3944	SMF 103891	Honduras	Lempira	MH449921

Norops morazani	JHT1638	MVZ:herp:257262	Honduras	Francisco Morazán	MH449708
Norops morazani	JHT1639	MVZ:herp:257263	Honduras	Francisco Morazán	MH449709
Norops morazani	JHT2040	UF 151772	Honduras	Francisco Morazán	MH449716
Norops morazani	JHT2041	SMF 87153	Honduras	Francisco Morazán	MH449717
Norops morazani	JHT2042	UF 151781	Honduras	Francisco Morazán	MH449718
Norops morazani	JHT2047	UF 151769	Honduras	Francisco Morazán	MH449719
Norops morazani	JHT2048	UF 151796	Honduras	Francisco Morazán	MH449720
Norops morazani	JHT2049	UF 151782	Honduras	Francisco Morazán	MH449721
Norops morazani	JHT2050	UF 151761	Honduras	Francisco Morazán	MH449722
Norops morazani	JHT2051	UF 151792	Honduras	Francisco Morazán	KU688007
Norops morazani	JHT2052	UF 151788	Honduras	Francisco Morazán	MH449723
Norops morazani	JHT2053	UF 151777	Honduras	Francisco Morazán	MH449724
Norops morazani	JHT2054	UF 151770	Honduras	Francisco Morazán	MH449725
Norops morazani	JHT2055	UF 151759	Honduras	Francisco Morazán	MH449726
Norops morazani	JHT2056	UF 151787	Honduras	Francisco Morazán	MH449727
Norops morazani	JHT2057	UF 151776	Honduras	Francisco Morazán	MH449728
Norops morazani	JHT2071	UF 151783	Honduras	Francisco Morazán	MH449729
Norops morazani	JHT2072	USNM 565040	Honduras	Francisco Morazán	MH449730
Norops morazani	JHT2073	UF 151775	Honduras	Francisco Morazán	MH449731
Norops morazani	JHT2074	MVZ:herp:257267	Honduras	Francisco Morazán	MH449732
Norops morazani	JHT2075	UF 151778	Honduras	Francisco Morazán	MH449733
Norops morazani	JHT2076	UF 151789	Honduras	Francisco Morazán	MH449734
Norops morazani	JHT2079	UF 151779	Honduras	Francisco Morazán	MH449735
Norops morazani	JHT2080	UF 151757	Honduras	Francisco Morazán	KU688009
Norops morazani	JHT2081	UF 151790	Honduras	Francisco Morazán	MH449736
Norops morazani	JHT2090	UF 151773	Honduras	Francisco Morazán	MH449737
Norops morazani	JHT2091	UF 151767	Honduras	Francisco Morazán	MH449738
Norops morazani	JHT2092	UF 151764	Honduras	Francisco Morazán	MH449739
Norops morazani	JHT2094	UF 151762	Honduras	Francisco Morazán	MH449740
Norops morazani	JHT2095	MVZ:herp:257268	Honduras	Francisco Morazán	MH449741
Norops morazani	JHT2096	UF 151795	Honduras	Francisco Morazán	MH449742
Norops morazani	JHT2097	UF 151784	Honduras	Francisco Morazán	KU688012
Norops morazani	JHT2098	UF 151791	Honduras	Francisco Morazán	KU688003
Norops morazani	JHT2099	UF 151760	Honduras	Francisco Morazán	MH449743
Norops morazani	JHT2100	UF 151756	Honduras	Francisco Morazán	MH449744
Norops morazani	JHT2101	-	Honduras	Francisco Morazán	MH449745
Norops morazani	JHT2102	-	Honduras	Francisco Morazán	MH449746
Norops morazani	JHT2103	UF 151785	Honduras	Francisco Morazán	MH449747

Norops morazani	JHT2104	UF 151758	Honduras	Francisco Morazán	KU688014
Norops morazani	JHT2105	UF 151774	Honduras	Francisco Morazán	MH449748
Norops morazani	JHT2106	UF 151763	Honduras	Francisco Morazán	MH449749
Norops morazani	JHT2107	UF 151793	Honduras	Francisco Morazán	MH449750
Norops morazani	JHT2108	UF 151767	Honduras	Francisco Morazán	KU688013
Norops morazani	JHT2109	UF 151771	Honduras	Francisco Morazán	MH449751
Norops morazani	JHT2110	UF 151768	Honduras	Francisco Morazán	MH449752
Norops morazani	JHT2111	UF 151765	Honduras	Francisco Morazán	KU688008
Norops morazani	JHT2112	UF 151794	Honduras	Francisco Morazán	MH449753
Norops morazani	JHT2966	UF 166239	Honduras	Francisco Morazán	MH449822
Norops morazani	JHT2967	UF 166240	Honduras	Francisco Morazán	MH449823
Norops morazani	JHT2968	UF 166241	Honduras	Francisco Morazán	KU688011
Norops morazani	JHT2969	UF 166242	Honduras	Francisco Morazán	KU688005
Norops morazani	JHT2970	UF 166243	Honduras	Francisco Morazán	MH449824
Norops morazani	JHT2971	UF 166244	Honduras	Francisco Morazán	MH449825
Norops morazani	JHT2972	UF 166245	Honduras	Francisco Morazán	MH449826
Norops morazani	JHT3188	USNM 578764	Honduras	Olancho	KU688010
Norops morazani	JHT3189	USNM 578765	Honduras	Olancho	KU688004
Norops morazani	JHT3190	USNM 578766	Honduras	Olancho	KU688006
Norops oscelloscapularis	JHT2845	UF 166246	Honduras	Copán	MH449816
Norops oxylophus	N087	UF 156044	Nicaragua	R.A.A. Norte	MH449963
Norops oxylophus	N590	UF 156046	Nicaragua	R.A.A. Norte	MH449968
Norops oxylophus	N608	UF 156047	Nicaragua	R.A.A. Norte	MH449969
Norops petersii	JHT1499	-	Honduras	Cortés	KU688015
Norops pijolense	JHT2358	UF 166247	Honduras	Yoro	MH449768
Norops pijolense	JHT2364	UF 166248	Honduras	Yoro	MH449769
Norops pijolense	JHT2365	UF 166249	Honduras	Yoro	KU688018
Norops pijolense	JHT2407	UF 166251	Honduras	Yoro	MH449789
Norops pijolense	JHT2408	UF 166252	Honduras	Yoro	KU688016
Norops pijolense	JHT2409	UF 166253	Honduras	Yoro	KU688020
Norops pijolense	JHT2410	UF 166254	Honduras	Yoro	MH449790
Norops pijolense	JHT2411	UF 166255	Honduras	Yoro	MH449791
Norops pijolense	JHT2412	UF 166256	Honduras	Yoro	MH449792
Norops pijolense	JHT2413	UF 166257	Honduras	Yoro	KU688017
Norops pijolense	JHT2414	UF 166258	Honduras	Yoro	MH449793
Norops pijolense	JHT2415	UF 166259	Honduras	Yoro	MH449794
Norops pijolense	JHT2789	UF 166260	Honduras	Yoro	MH449813
Norops pijolense	JHT2790	UF 166261	Honduras	Yoro	KU688019

Norops purpurgularis	JHT2444	UF 166263	Honduras	Yoro	KU688021
Norops quaggulus	JHT2281	-	Nicaragua	Matagalpa	MH449758
Norops quaggulus	JHT2282	-	Nicaragua	Matagalpa	MH449759
Norops quaggulus	N273	-	Nicaragua	Jinotega	MH449967
Norops rodriguezii	JHT2355	UF 166213	Honduras	Santa Bárbara	MH449767
Norops rodriguezii	JHT2586	UF 166238	Honduras	Santa Bárbara	MH449810
Norops rodriguezii	JHT3708	CM 161345	Honduras	Lempira	MH449901
Norops rodriguezii	JHT3709	CM 161346	Honduras	Lempira	MH449902
Norops rodriguezii	JHT3710	CM 161347	Honduras	Lempira	MH449903
Norops rodriguezii	JHT3711	CM 161348	Honduras	Lempira	MH449904
Norops rodriguezii	LDW11424	-	Honduras	Cortés	MH449935
Norops rubribarbaris	JHT2342	UF 152661	Honduras	Santa Bárbara	KU688023
Norops rubribarbaris	JHT2343	UF 152660	Honduras	Santa Bárbara	KU688024
Norops rubribarbaris	JHT2344	UF 152662	Honduras	Santa Bárbara	KU688025
Norops rubribarbaris	JHT3224	USNM 578804	Honduras	Atlántida	MH449856
Norops rubribarbaris	JHT3323	USNM 578761	Honduras	Santa Barbara	KU688022
Norops rubribarbaris	JHT2623	UF 166189	Honduras	La Paz	KU687948
Norops rubribarbaris	JHT2876	-	Honduras	La Paz	KU687953
Norops serranoi	-	KU 291367	El Salvador	La Libertad	MH449934
Norops sminthus	JHT2289	UF 166266	Honduras	Comayagua	KU688028
Norops sminthus	JHT2290	UF 166267	Honduras	Comayagua	KU688029
Norops sminthus	JHT2291	UF 166268	Honduras	Comayagua	KU688026
Norops sminthus	JHT2292	UF 166269	Honduras	Comayagua	KU688027
Norops sminthus	JHT2293	UF 166270	Honduras	Comayagua	KU688030
Norops sminthus	JHT2295	UF 166272	Honduras	Comayagua	MH449760
Norops sminthus	JHT2296	UF 166273	Honduras	Comayagua	KU688033
Norops sminthus	JHT2297	UF 166274	Honduras	Comayagua	KU688034
Norops sminthus	JHT2298	UF 166275	Honduras	Comayagua	KU688032
Norops sminthus	JHT2301	UF 166276	Honduras	Comayagua	MF094504
Norops sminthus	JHT2302	UF 166277	Honduras	Comayagua	KU688031
Norops uniformis	GK5228	SMF 99541	Mexico	Veracruz	MH449974
Norops uniformis	GK5229	SMF 99542	Mexico	Veracruz	MH449975
Norops uniformis	GK5316	SMF 99546	Mexico	Veracruz	MH449976
Norops uniformis	GK5538	SMF 100139	Mexico	Chiapas	MH449977
Norops uniformis	GK5192	SMF 99539	Mexico	Veracruz	MH449973
Norops unilobatus	JHT2348	UF 166264	Honduras	Santa Bárbara	MH449765
Norops unilobatus	JHT3352	CM 161322	Honduras	Cortes	MH449865
Norops unilobatus	JHT3353	CM 161323	Honduras	Cortes	MH449866

Norops wellbornae	JHT3521	CM 161332	Honduras	Valle	MH449885
Norops wellbornae	JHT3522	CM 161333	Honduras	Valle	MH449886
Norops wellbornae	JHT3523	CM 161334	Honduras	Valle	MH449887
Norops wellbornae	JHT3524	CM 161335	Honduras	Valle	MH449888
Norops wellbornae	JHT3555	CM 161336	Honduras	Valle	MH449889
Norops wellbornae	JHT3580	CM 161337	Honduras	Valle	MH449890
Norops wellbornae	JHT3581	CM 161338	Honduras	Valle	MH449891
Norops wellbornae	JHT3597	CM 161339	Honduras	Valle	MH449892
Norops wellbornae	JHT3598	CM 161340	Honduras	Valle	MH449893
Norops wellbornae	JHT3606	CM 161342	Honduras	Valle	MH449894
Norops wermuthi	N562	UF 156202	Nicaragua	Jinotega	KU688037
Norops wermuthi	N956	UF 156206	Nicaragua	Jinotega	KU688036
Norops wilsoni	CAC048	USNM 578773	Honduras	Atlántida	MH449694
Norops wilsoni	CAC050	USNM 578774	Honduras	Atlántida	MH449695
Norops wilsoni	JHT3361	SMF 100103	Honduras	Atlántida	MH449869
Norops wilsoni	MMF204	SMF 100102	Honduras	Atlántida	MH449959
Norops yoroensis	JHT2032	SMF 87164	Honduras	Francisco Morazán	MH449711
Norops yoroensis	JHT2033	SMF 87165	Honduras	Francisco Morazán	MH449712
Norops yoroensis	JHT2034	SMF 87166	Honduras	Francisco Morazán	MH449713
Norops yoroensis	JHT2367	UF 166309	Honduras	Yoro	MH449770
Norops yoroensis	JHT2368	UF 166310	Honduras	Yoro	MH449771
Norops yoroensis	JHT2391	UF 166313	Honduras	Yoro	MH449779
Norops yoroensis	JHT2393	UF 166315	Honduras	Yoro	MH449780
Norops yoroensis	JHT2398	UF 166316	Honduras	Yoro	MH449781
Norops yoroensis	JHT2399	UF 166317	Honduras	Yoro	MH449782
Norops yoroensis	JHT2400	UF 166318	Honduras	Yoro	MH449783
Norops yoroensis	JHT2402	UF 166320	Honduras	Yoro	MH449784
Norops yoroensis	JHT2403	UF 166321	Honduras	Yoro	MH449785
Norops yoroensis	JHT2404	UF 166322	Honduras	Yoro	MH449786
Norops yoroensis	JHT2405	UF 166323	Honduras	Yoro	MH449787
Norops yoroensis	JHT2406	UF 166324	Honduras	Yoro	MH449788
Norops yoroensis	JHT2784	UF 166328	Honduras	Yoro	MH449811
Norops yoroensis	JHT2785	UF 166329	Honduras	Yoro	MH449812
Norops yoroensis	JHT2792	UF 166330	Honduras	Yoro	MH449814
Norops yoroensis	JHT2794	UF 166332	Honduras	Yoro	MH449815
Norops yoroensis	JHT2942	-	Honduras	Yoro	MH449817
Norops yoroensis	JHT2943	UF 166333	Honduras	Yoro	MH449818
Norops yoroensis	JHT2944	UF 166334	Honduras	Yoro	MH449819

Norops yoroensis	JHT2948	UF 166335	Honduras	Yoro	MH449820
Norops yoroensis	CAC011	USNM 578810	Honduras	Atlántida	MH449688
Norops yoroensis	CAC020	USNM 578813	Honduras	Atlántida	MH449689
Norops yoroensis	CAC021	USNM 578814	Honduras	Atlántida	MH449690
Norops yoroensis	CAC022	USNM 578815	Honduras	Atlántida	MH449692
Norops yoroensis	JHT3044	USNM 578820	Honduras	Atlántida	MH449834
Norops yoroensis	JHT3052	USNM 578779	Honduras	Atlántida	MH449836
Norops yoroensis	JHT3053	USNM 578780	Honduras	Atlántida	MH449837
Norops yoroensis	JHT3054	USNM 578781	Honduras	Atlántida	MH449838
Norops yoroensis	JHT3086	USNM 578782	Honduras	Atlántida	MH449839
Norops yoroensis	JHT3087	USNM 578783	Honduras	Atlántida	MH449340
Norops yoroensis	JHT3088	USNM 578784	Honduras	Atlántida	MH449841
Norops yoroensis	JHT3089	USNM 578785	Honduras	Atlántida	MH449842
Norops yoroensis	JHT3092	USNM 578788	Honduras	Atlántida	MH449843
Norops yoroensis	JHT3093	USNM 578789	Honduras	Atlántida	MH449844
Norops yoroensis	JHT3132	USNM 578797	Honduras	Atlántida	MH449846
Norops yoroensis	JHT3133	USNM 578798	Honduras	Atlántida	MH449847
Norops yoroensis	JHT3140	USNM 578799	Honduras	Atlántida	MH449848
Norops yoroensis	JHT3156	USNM 578800	Honduras	Atlántida	MH449849
Norops yoroensis	JHT3162	USNM 578801	Honduras	Atlántida	MH449850
Norops yoroensis	JHT3163	USNM 578802	Honduras	Atlántida	MH449851
Norops yoroensis	JHT3164	USNM 578803	Honduras	Atlántida	MH449852
Norops yoroensis	JHT3225	USNM 578805	Honduras	Atlántida	MH449857
Norops yoroensis	JHT3226	USNM 578806	Honduras	Atlántida	MH449858
Norops yoroensis	JHT3227	USNM 578807	Honduras	Atlántida	MH449859
Norops yoroensis	JHT3228	USNM 578808	Honduras	Atlántida	MH449860
Norops yoroensis	JHT3249	USNM 578809	Honduras	Atlántida	MH449861
Norops yoroensis	LDW13136	SMF 88701	Honduras	Atlántida	MH449936
Norops yoroensis	MMF198	CM 161382	Honduras	Atlántida	MH449953
Norops yoroensis	MMF199	CM 161383	Honduras	Atlántida	MH449954
Norops yoroensis	MMF200	CM 161384	Honduras	Atlántida	MH449955
Norops yoroensis	MMF201	CM 161385	Honduras	Atlántida	MH449956
Norops yoroensis	MMF202	CM 161386	Honduras	Atlántida	MH449957
Norops yoroensis	MMF203	CM 161387	Honduras	Atlántida	MH449958
Norops yoroensis	MMF206	CM 161388	Honduras	Atlántida	MH449960
Norops yoroensis	MMF208	CM 161389	Honduras	Atlántida	MH449962
Norops yoroensis	JHT1415	UF 149654	Honduras	Cortés	MH449704
Norops zeus	IRL061	UF 166181	Honduras	Cortés	MH449698

Norops zeus	JHT2378	UF 166231	Honduras	Cortés	MH449772
Norops zeus	JHT2379	UF 166232	Honduras	Cortés	MH449773
Norops zeus	JHT2380	UF 166233	Honduras	Cortés	MH449774
Norops zeus	JHT2381	UF 166234	Honduras	Cortés	MH449775
Norops zeus	JHT2515	UF 166237	Honduras	Cortés	MH449805
Norops zeus	JHT2980	CM 161316	Honduras	Cortés	MH449827
Norops zeus	JHT2981	CM 161317	Honduras	Cortés	MH449828
Norops zeus	JHT2982	CM 161318	Honduras	Cortés	MH449829
Norops zeus	JHT3025	CM 161319	Honduras	Atlántida	MH449832
Norops zeus	JHT3036	USNM 578818	Honduras	Atlántida	MH449833
Norops zeus	JHT3343	CM 161321	Honduras	Yoro	MH449863
Norops zeus	JHT3344	CM 161320	Honduras	Yoro	MH449864
Norops zeus	JHT3359	-	Honduras	Atlántida	MH449867
Norops zeus	JHT3360	CM 161324	Honduras	Atlántida	MH449868

Supplementary Table 2. Results of substitution saturation tests (Xia and Lemey, 2009).

Codon position	NumOTUs	Iss	Iss.cSym	Iss.cSym P	Iss.cAsym	Iss.cAsym P
**All codon positions**	4	0.297	0.805	0.0000	0.744	0.0000
	8	0.300	0.765	0.0000	0.656	0.0000
	16	0.298	0.744	0.0000	0.534	0.0000
	32	0.305	0.718	0.0000	0.392	0.0012
**Codon position 1 & 2**	4	0.084	0.791	0.0000	0.758	0.0000
	8	0.094	0.745	0.0000	0.634	0.0000
	16	0.098	0.709	0.0000	0.499	0.0000
	32	0.111	0.695	0.0000	0.367	0.0000
**Codon position 3**	4	0.604	0.777	0.0000	0.764	0.0000
	8	0.604	0.733	0.0002	0.633	0.3940
	16	0.602	0.650	0.1498	0.457	0.0000
	32	0.606	0.687	0.0159	0.367	0.0000

Supplementary Table 3. Assignment of specimens to clusters (given as cluster number) based on all analyses of the full dataset (412 sequences). Specimens are labelled with either Field or Museum IDs.

ID	*Genus*	*species*	ABGD 10%	ABGD 7.2%	ABGD 5.2%	BOLD RESL	mPTP	bPTP	GMYC
CAC005	*Norops*	*mccraniei*	1	1	1	23	7	22	1
CAC043	*Norops*	*mccraniei*	1	1	1	23	7	22	1
JHT1402	*Norops*	*mccraniei*	1	1	1	23	7	22	1
JHT1403	*Norops*	*mccraniei*	1	1	1	23	7	22	1
JHT1404	*Norops*	*mccraniei*	1	1	1	23	7	22	1
JHT1412	*Norops*	*mccraniei*	1	1	1	23	7	22	1
JHT1414	*Norops*	*mccraniei*	1	1	1	23	7	22	1
JHT1699	*Norops*	*mccraniei*	1	1	1	23	7	22	1
JHT2037	*Norops*	*mccraniei*	1	1	1	23	7	22	1
JHT2038	*Norops*	*mccraniei*	1	1	1	23	7	22	1
JHT2127	*Norops*	*mccraniei*	1	1	1	23	7	22	1
JHT2147	*Norops*	*mccraniei*	1	1	1	23	7	22	1
JHT2303	*Norops*	*mccraniei*	1	1	1	23	7	22	1
JHT2354	*Norops*	*mccraniei*	1	1	1	23	7	22	1
JHT2424	*Norops*	*mccraniei*	1	1	1	23	7	22	1
JHT2425	*Norops*	*mccraniei*	1	1	1	23	7	22	1
JHT2460	*Norops*	*mccraniei*	1	1	1	23	7	22	1
JHT2462	*Norops*	*mccraniei*	1	1	1	23	7	22	1
JHT2962	*Norops*	*mccraniei*	1	1	1	23	7	22	1
JHT3006	*Norops*	*mccraniei*	1	1	1	23	7	22	1
JHT3365	*Norops*	*mccraniei*	1	1	1	23	7	22	1
JHT3366	*Norops*	*mccraniei*	1	1	1	23	7	22	1
JHT3367	*Norops*	*mccraniei*	1	1	1	23	7	22	1
JHT3393	*Norops*	*mccraniei*	1	1	1	23	7	22	1
JHT3394	*Norops*	*mccraniei*	1	1	1	23	7	22	1
JHT3395	*Norops*	*mccraniei*	1	1	1	23	7	22	1

JHT3413	*Norops*	*mccraniei*	1	1	1	23	7	22	1
JHT3414	*Norops*	*mccraniei*	1	1	1	23	7	22	1
JHT3415	*Norops*	*mccraniei*	1	1	1	23	7	22	1
JHT3930	*Norops*	*mccraniei*	1	1	1	23	7	22	1
MMF132	*Norops*	*mccraniei*	1	1	1	23	7	22	1
MMF146	*Norops*	*mccraniei*	1	1	1	23	7	22	1
MMF148	*Norops*	*mccraniei*	1	1	1	23	7	22	1
JHT3633	*Norops*	*mccraniei (aff)*	1	35	35	26	6	21	2
JHT3634	*Norops*	*mccraniei (aff)*	1	35	35	26	6	21	2
JHT3652	*Norops*	*mccraniei (aff)*	1	35	35	26	6	21	2
JHT3653	*Norops*	*mccraniei (aff)*	1	35	35	26	6	21	2
JHT3663	*Norops*	*mccraniei (aff)*	1	35	35	26	6	21	2
JHT3943	*Norops*	*mccraniei (aff)*	1	35	35	26	6	21	2
JHT3944	*Norops*	*mccraniei (aff)*	1	35	35	26	6	21	2
CAC011	*Norops*	*yoroensis*	2	2	2	44	18	11	8
CAC020	*Norops*	*yoroensis*	2	2	2	44	18	11	8
CAC021	*Norops*	*yoroensis*	2	2	2	44	18	11	8
CAC022	*Norops*	*yoroensis*	2	2	2	44	18	11	8
JHT3044	*Norops*	*yoroensis*	2	2	2	44	18	11	8
JHT3052	*Norops*	*yoroensis*	2	2	2	44	18	11	8
JHT3053	*Norops*	*yoroensis*	2	2	2	44	18	11	8
JHT3054	*Norops*	*yoroensis*	2	2	2	44	18	11	8
JHT3086	*Norops*	*yoroensis*	2	2	2	44	18	11	8
JHT3087	*Norops*	*yoroensis*	2	2	2	44	18	11	8
JHT3088	*Norops*	*yoroensis*	2	2	2	44	18	11	8
JHT3089	*Norops*	*yoroensis*	2	2	2	44	18	11	8
JHT3092	*Norops*	*yoroensis*	2	2	2	44	18	11	8
JHT3093	*Norops*	*yoroensis*	2	2	2	44	18	11	8
JHT3132	*Norops*	*yoroensis*	2	2	2	44	18	11	8
JHT3133	*Norops*	*yoroensis*	2	2	2	44	18	11	8
JHT3140	*Norops*	*yoroensis*	2	2	2	44	18	11	8

JHT3156	*Norops*	*yoroensis*	2	2	2	44	18	11	8
JHT3162	*Norops*	*yoroensis*	2	2	2	44	18	11	8
JHT3163	*Norops*	*yoroensis*	2	2	2	44	18	11	8
JHT3164	*Norops*	*yoroensis*	2	2	2	44	18	11	8
JHT3225	*Norops*	*yoroensis*	2	2	2	44	18	11	8
JHT3226	*Norops*	*yoroensis*	2	2	2	44	18	11	8
JHT3227	*Norops*	*yoroensis*	2	2	2	44	18	11	8
JHT3228	*Norops*	*yoroensis*	2	2	2	44	18	11	8
JHT3249	*Norops*	*yoroensis*	2	2	2	44	18	11	8
LDW13136	*Norops*	*yoroensis*	2	2	2	44	18	11	8
MMF198	*Norops*	*yoroensis*	2	2	2	44	18	11	8
MMF199	*Norops*	*yoroensis*	2	2	2	44	18	11	8
MMF200	*Norops*	*yoroensis*	2	2	2	44	18	11	8
MMF201	*Norops*	*yoroensis*	2	2	2	44	18	11	8
MMF202	*Norops*	*yoroensis*	2	2	2	44	18	11	8
MMF203	*Norops*	*yoroensis*	2	2	2	44	18	11	8
MMF206	*Norops*	*yoroensis*	2	2	2	44	18	11	8
MMF208	*Norops*	*yoroensis*	2	2	2	44	18	11	8
CAC025	*Norops*	*lemurinus*	3	3	3	19	13	52	10
IRL022	*Norops*	*lemurinus*	3	3	3	22	14	53	11
IRL032	*Norops*	*lemurinus*	3	3	3	22	14	53	11
JHT2346	*Norops*	*lemurinus*	3	3	3	22	14	53	11
JHT2347	*Norops*	*lemurinus*	3	3	3	22	14	53	11
JHT2382	*Norops*	*lemurinus*	3	3	3	22	14	53	11
JHT2383	*Norops*	*lemurinus*	3	3	3	22	14	53	11
JHT2384	*Norops*	*lemurinus*	3	3	3	22	14	53	11
JHT2484	*Norops*	*lemurinus*	3	3	3	22	14	53	11
JHT2490	*Norops*	*lemurinus*	3	3	3	22	14	53	11
JHT2491	*Norops*	*lemurinus*	3	3	3	22	14	53	11
JHT2495	*Norops*	*lemurinus*	3	3	3	22	14	53	11
JHT2500	*Norops*	*lemurinus*	3	3	3	22	14	53	11

JHT2514	*Norops*	*lemurinus*	3	3	3	22	14	53	11
JHT2584	*Norops*	*lemurinus*	3	3	3	22	14	53	11
JHT2585	*Norops*	*lemurinus*	3	3	3	22	14	53	11
JHT3023	*Norops*	*lemurinus*	3	3	3	22	14	53	11
JHT3202	*Norops*	*lemurinus*	3	3	3	22	14	53	11
JHT3203	*Norops*	*lemurinus*	3	3	3	22	14	53	11
JHT3204	*Norops*	*lemurinus*	3	3	3	22	14	53	11
JHT3291	*Norops*	*lemurinus*	3	3	3	22	14	53	11
JHT3422	*Norops*	*lemurinus*	3	3	3	19	13	52	10
CAC048	*Norops*	*wilsoni*	4	4	4	43	5	20	3
CAC050	*Norops*	*wilsoni*	4	4	4	43	5	20	3
JHT3361	*Norops*	*wilsoni*	4	4	4	43	5	20	3
MMF204	*Norops*	*wilsoni*	4	4	4	43	5	20	3
JHT3488	*Norops*	*limifrons*	5	5	36	21	9	26	14
JHT3959	*Norops*	*limifrons*	5	5	36	21	9	26	14
JHT3960	*Norops*	*limifrons*	5	5	36	21	9	26	14
JHT3965	*Norops*	*limifrons*	5	5	36	21	9	26	14
JHT3978	*Norops*	*limifrons*	5	5	36	21	9	26	14
JHT3979	*Norops*	*limifrons*	5	5	36	21	9	26	14
JHT3980	*Norops*	*limifrons*	5	5	36	21	9	26	14
JHT3981	*Norops*	*limifrons*	5	5	36	21	9	26	14
JHT3982	*Norops*	*limifrons*	5	5	36	21	9	26	14
JHT3983	*Norops*	*limifrons*	5	5	36	21	9	26	14
JHT4004	*Norops*	*limifrons*	5	5	36	21	9	26	14
IRL061	*Norops*	*zeus*	5	5	5	48	10	27	13
JHT2378	*Norops*	*zeus*	5	5	5	48	10	27	13
JHT2379	*Norops*	*zeus*	5	5	5	48	10	27	13
JHT2380	*Norops*	*zeus*	5	5	5	48	10	27	13
JHT2381	*Norops*	*zeus*	5	5	5	48	10	27	13
JHT2515	*Norops*	*zeus*	5	5	5	48	10	27	13
JHT2980	*Norops*	*zeus*	5	5	5	48	10	27	13

JHT2981	*Norops*	*zeus*	5	5	5	48	10	27	13
JHT2982	*Norops*	*zeus*	5	5	5	48	10	27	13
JHT3025	*Norops*	*zeus*	5	5	5	48	10	27	13
JHT3036	*Norops*	*zeus*	5	5	5	48	10	27	13
JHT3343	*Norops*	*zeus*	5	5	5	48	10	27	13
JHT3344	*Norops*	*zeus*	5	5	5	48	10	27	13
JHT3359	*Norops*	*zeus*	5	5	5	48	10	27	13
JHT3360	*Norops*	*zeus*	5	5	5	48	10	27	13
JHT1301	*Norops*	*cusuco*	6	6	6	10	22	19	17
JHT2927	*Norops*	*cusuco*	6	6	6	10	22	19	17
JHT2983	*Norops*	*cusuco*	6	6	6	10	22	19	17
JHT2984	*Norops*	*cusuco*	6	6	6	10	22	19	17
JHT2985	*Norops*	*cusuco*	6	6	6	10	22	19	17
JHT1415	*Norops*	*yoroensis*	7	7	7	49	16	28	42
JHT2032	*Norops*	*yoroensis*	7	7	7	50	17	29	9
JHT2033	*Norops*	*yoroensis*	7	7	7	50	17	29	9
JHT2034	*Norops*	*yoroensis*	7	7	7	50	17	29	9
JHT2367	*Norops*	*yoroensis*	7	7	7	50	17	29	9
JHT2368	*Norops*	*yoroensis*	7	7	7	50	17	29	9
JHT2391	*Norops*	*yoroensis*	7	7	7	50	17	29	9
JHT2393	*Norops*	*yoroensis*	7	7	7	50	17	29	9
JHT2398	*Norops*	*yoroensis*	7	7	7	50	17	29	9
JHT2399	*Norops*	*yoroensis*	7	7	7	50	17	29	9
JHT2400	*Norops*	*yoroensis*	7	7	7	50	17	29	9
JHT2402	*Norops*	*yoroensis*	7	7	7	50	17	29	9
JHT2403	*Norops*	*yoroensis*	7	7	7	50	17	29	9
JHT2404	*Norops*	*yoroensis*	7	7	7	50	17	29	9
JHT2405	*Norops*	*yoroensis*	7	7	7	50	17	29	9
JHT2406	*Norops*	*yoroensis*	7	7	7	50	17	29	9
JHT2784	*Norops*	*yoroensis*	7	7	7	50	17	29	9
JHT2785	*Norops*	*yoroensis*	7	7	7	50	17	29	9

JHT2792	*Norops*	*yoroensis*	7	7	7	50	17	29	9
JHT2794	*Norops*	*yoroensis*	7	7	7	50	17	29	9
JHT2942	*Norops*	*yoroensis*	7	7	7	50	17	29	9
JHT2943	*Norops*	*yoroensis*	7	7	7	50	17	29	9
JHT2944	*Norops*	*yoroensis*	7	7	7	50	17	29	9
JHT2948	*Norops*	*yoroensis*	7	7	7	50	17	29	9
JHT1499	*Norops*	*petersii*	8	8	8	29	33	4	46
JHT1586	*Norops*	*johnmeyeri*	9	9	9	16	30	5	27
JHT1587	*Norops*	*johnmeyeri*	9	9	9	16	30	5	27
JHT1594	*Norops*	*johnmeyeri*	9	9	9	16	30	5	27
JHT1595	*Norops*	*johnmeyeri*	9	9	9	16	30	5	27
JHT1605	*Norops*	*johnmeyeri*	9	9	9	16	30	5	27
JHT1606	*Norops*	*johnmeyeri*	9	9	9	16	30	5	27
JHT1607	*Norops*	*johnmeyeri*	9	9	9	16	30	5	27
JHT1596	*Norops*	*amplisquamosus*	10	10	10	2	36	3	30
JHT1602	*Norops*	*amplisquamosus*	10	10	10	2	36	3	30
JHT1615	*Norops*	*amplisquamosus*	10	10	10	2	36	3	30
JHT1620	*Norops*	*amplisquamosus*	10	10	10	2	36	3	30
JHT1621	*Norops*	*amplisquamosus*	10	10	10	2	36	3	30
JHT2986	*Norops*	*amplisquamosus*	10	10	10	2	36	3	30
JHT1638	*Norops*	*morazani*	11	11	11	25	46	24	31
JHT1639	*Norops*	*morazani*	11	11	11	25	46	24	31
JHT2040	*Norops*	*morazani*	11	11	11	25	46	24	31
JHT2041	*Norops*	*morazani*	11	11	11	25	46	24	31
JHT2042	*Norops*	*morazani*	11	11	11	25	46	24	31
JHT2047	*Norops*	*morazani*	11	11	11	25	46	24	31
JHT2048	*Norops*	*morazani*	11	11	11	25	46	24	31
JHT2049	*Norops*	*morazani*	11	11	11	25	46	24	31
JHT2050	*Norops*	*morazani*	11	11	11	25	46	24	31
JHT2051	*Norops*	*morazani*	11	11	11	25	46	24	31
JHT2052	*Norops*	*morazani*	11	11	11	25	46	24	31

JHT2053	*Norops*	*morazani*	11	11	11	25	46	24	31
JHT2054	*Norops*	*morazani*	11	11	11	25	46	24	31
JHT2055	*Norops*	*morazani*	11	11	11	25	46	24	31
JHT2056	*Norops*	*morazani*	11	11	11	25	46	24	31
JHT2057	*Norops*	*morazani*	11	11	11	25	46	24	31
JHT2071	*Norops*	*morazani*	11	11	11	25	46	24	31
JHT2072	*Norops*	*morazani*	11	11	11	25	46	24	31
JHT2073	*Norops*	*morazani*	11	11	11	25	46	24	31
JHT2074	*Norops*	*morazani*	11	11	11	25	46	24	31
JHT2075	*Norops*	*morazani*	11	11	11	25	46	24	31
JHT2076	*Norops*	*morazani*	11	11	11	25	46	24	31
JHT2079	*Norops*	*morazani*	11	11	11	25	46	24	31
JHT2080	*Norops*	*morazani*	11	11	11	25	46	24	31
JHT2081	*Norops*	*morazani*	11	11	11	25	46	24	31
JHT2090	*Norops*	*morazani*	11	11	11	25	46	24	31
JHT2091	*Norops*	*morazani*	11	11	11	25	46	24	31
JHT2092	*Norops*	*morazani*	11	11	11	25	46	24	31
JHT2094	*Norops*	*morazani*	11	11	11	25	46	24	31
JHT2095	*Norops*	*morazani*	11	11	11	25	46	24	31
JHT2096	*Norops*	*morazani*	11	11	11	25	46	24	31
JHT2097	*Norops*	*morazani*	11	11	11	25	46	24	31
JHT2098	*Norops*	*morazani*	11	11	11	25	46	24	31
JHT2099	*Norops*	*morazani*	11	11	11	25	46	24	31
JHT2100	*Norops*	*morazani*	11	11	11	25	46	24	31
JHT2101	*Norops*	*morazani*	11	11	11	25	46	24	31
JHT2102	*Norops*	*morazani*	11	11	11	25	46	24	31
JHT2103	*Norops*	*morazani*	11	11	11	25	46	24	31
JHT2104	*Norops*	*morazani*	11	11	11	25	46	24	31
JHT2105	*Norops*	*morazani*	11	11	11	25	46	24	31
JHT2106	*Norops*	*morazani*	11	11	11	25	46	24	31
JHT2107	*Norops*	*morazani*	11	11	11	25	46	24	31

JHT2108	*Norops*	*morazani*	11	11	11	25	46	24	31
JHT2109	*Norops*	*morazani*	11	11	11	25	46	24	31
JHT2110	*Norops*	*morazani*	11	11	11	25	46	24	31
JHT2111	*Norops*	*morazani*	11	11	11	25	46	24	31
JHT2112	*Norops*	*morazani*	11	11	11	25	46	24	31
JHT2966	*Norops*	*morazani*	11	11	11	25	46	24	31
JHT2967	*Norops*	*morazani*	11	11	11	25	46	24	31
JHT2968	*Norops*	*morazani*	11	11	11	25	46	24	31
JHT2969	*Norops*	*morazani*	11	11	11	25	46	24	31
JHT2970	*Norops*	*morazani*	11	11	11	25	46	24	31
JHT2971	*Norops*	*morazani*	11	11	11	25	46	24	31
JHT2972	*Norops*	*morazani*	11	11	11	25	46	24	31
JHT3188	*Norops*	*morazani (aff)*	11	36	37	28	45	23	32
JHT3189	*Norops*	*morazani (aff)*	11	36	37	28	45	23	32
JHT3190	*Norops*	*morazani (aff)*	11	36	37	28	45	23	32
JHT2000	*Norops*	*laeviventris*	12	12	12	18	20	51	19
JHT2152	*Norops*	*laeviventris*	12	12	12	18	20	51	19
JHT2229	*Norops*	*laeviventris*	12	12	12	18	20	51	19
JHT2278	*Norops*	*laeviventris*	12	12	12	17	20	50	20
JHT2284	*Norops*	*laeviventris*	12	12	12	17	20	50	20
JHT2320	*Norops*	*laeviventris*	12	12	12	18	20	51	19
JHT2394	*Norops*	*laeviventris*	12	12	12	20	19	34	45
JHT2532	*Norops*	*laeviventris*	12	12	12	18	20	51	19
JHT2533	*Norops*	*laeviventris*	12	12	12	18	20	51	19
JHT2542	*Norops*	*laeviventris*	12	12	12	18	20	51	19
JHT2543	*Norops*	*laeviventris*	12	12	12	18	20	51	19
JHT2545	*Norops*	*laeviventris*	12	12	12	18	20	51	19
JHT2973	*Norops*	*laeviventris*	12	12	12	18	20	51	19
JHT3390	*Norops*	*laeviventris*	12	12	12	18	20	51	19
JHT3635	*Norops*	*laeviventris*	12	12	12	18	20	51	19
JHT3916	*Norops*	*laeviventris*	12	12	12	18	20	51	19

JHT4005	*Norops*	*laeviventris*	12	12	12	17	20	50	20
MMF170	*Norops*	*laeviventris*	12	12	12	18	20	51	19
MMF171	*Norops*	*laeviventris*	12	12	12	18	20	51	19
JHT2266	*Norops*	*capito*	13	13	13	5	4	9	4
JHT2267	*Norops*	*capito*	13	13	13	5	4	9	4
JHT3416	*Norops*	*capito*	13	13	13	5	4	9	4
MMF113	*Norops*	*capito*	13	13	13	5	4	9	4
MMF133	*Norops*	*capito*	13	13	13	5	4	9	4
N1016	*Norops*	*capito*	13	13	13	5	4	9	4
N117	*Norops*	*capito*	13	13	13	5	4	9	4
N266	*Norops*	*capito*	13	13	13	5	4	9	4
JHT2281	*Norops*	*quaggulus*	14	14	14	31	2	2	5
JHT2282	*Norops*	*quaggulus*	14	14	14	31	2	2	5
N273	*Norops*	*quaggulus*	14	14	14	31	2	2	5
JHT2289	*Norops*	*sminthus*	15	15	15	41	44	15	33
JHT2290	*Norops*	*sminthus*	15	15	15	41	44	15	33
JHT2291	*Norops*	*sminthus*	15	15	15	41	44	15	33
JHT2292	*Norops*	*sminthus*	15	15	15	41	44	15	33
JHT2293	*Norops*	*sminthus*	15	15	15	41	44	15	33
JHT2295	*Norops*	*sminthus*	15	15	15	41	44	15	33
JHT2296	*Norops*	*sminthus*	15	15	15	41	44	15	33
JHT2297	*Norops*	*sminthus*	15	15	15	41	44	15	33
JHT2298	*Norops*	*sminthus*	15	15	15	41	44	15	33
JHT2301	*Norops*	*sminthus*	15	15	15	41	44	15	33
JHT2302	*Norops*	*sminthus*	15	15	15	41	44	15	33
JHT2317	*Norops*	*heteropholidotus*	16	16	16	13	42	49	35
JHT2318	*Norops*	*heteropholidotus*	16	16	16	13	42	49	35
JHT2319	*Norops*	*heteropholidotus*	16	16	16	13	42	49	35
JHT2321	*Norops*	*heteropholidotus*	16	16	16	13	42	49	35
JHT2322	*Norops*	*heteropholidotus*	16	16	16	13	42	49	35
JHT2323	*Norops*	*heteropholidotus*	16	16	16	13	42	49	35

JHT2325	*Norops*	*heteropholidotus*	16	16	16	13	42	49	35
JHT2326	*Norops*	*heteropholidotus*	16	16	16	13	42	49	35
JHT2702	*Norops*	*heteropholidotus*	16	16	38	12	40	46	36
JHT2703	*Norops*	*heteropholidotus*	16	16	38	12	40	46	36
JHT2704	*Norops*	*heteropholidotus*	16	16	38	12	40	46	36
JHT2705	*Norops*	*heteropholidotus*	16	16	38	12	40	46	36
JHT2706	*Norops*	*heteropholidotus*	16	16	38	12	40	46	36
JHT2707	*Norops*	*heteropholidotus*	16	16	38	12	40	46	36
JHT2708	*Norops*	*heteropholidotus*	16	16	38	12	40	46	36
JHT2709	*Norops*	*heteropholidotus*	16	16	38	12	40	46	36
JHT2710	*Norops*	*heteropholidotus*	16	16	38	12	40	46	36
JHT2711	*Norops*	*heteropholidotus*	16	16	38	12	40	46	36
JHT2712	*Norops*	*heteropholidotus*	16	16	38	12	40	46	36
JHT2721	*Norops*	*heteropholidotus*	16	16	38	12	40	46	36
JHT2722	*Norops*	*heteropholidotus*	16	16	38	12	40	46	36
JHT2723	*Norops*	*heteropholidotus*	16	16	38	12	40	46	36
JHT2885	*Norops*	*heteropholidotus*	16	16	16	13	42	49	35
JHT2893	*Norops*	*heteropholidotus*	16	16	16	13	42	49	35
JHT3639	*Norops*	*heteropholidotus*	16	16	39	14	41	48	49
JHT3763	*Norops*	*heteropholidotus*	16	16	16	13	42	49	35
JHT3817	*Norops*	*heteropholidotus*	16	16	16	13	42	49	35
JHT3818	*Norops*	*heteropholidotus*	16	16	16	13	42	49	35
JHT3821	*Norops*	*heteropholidotus*	16	16	16	13	42	49	35
JHT3891	*Norops*	*heteropholidotus*	16	16	16	13	42	49	35
JHT3892	*Norops*	*heteropholidotus*	16	16	16	13	42	49	35
JHT3893	*Norops*	*heteropholidotus*	16	16	16	13	42	49	35
JHT3903	*Norops*	*heteropholidotus*	16	16	16	13	42	49	35
KU291251	*Norops*	*heteropholidotus*	16	16	38	12	40	46	36
MMF005	*Norops*	*heteropholidotus*	16	16	40	11	39	45	37
MMF006	*Norops*	*heteropholidotus*	16	16	40	11	39	45	37
JHT2342	*Norops*	*rubribarbaris*	17	17	17	38	38	13	38

JHT2343	*Norops*	*rubribarbaris*	17	17	17	38	38	13	38
JHT2344	*Norops*	*rubribarbaris*	17	17	17	38	38	13	38
JHT3224	*Norops*	*rubribarbaris*	17	17	17	38	38	13	38
JHT3323	*Norops*	*rubribarbaris*	17	17	17	38	38	13	38
JHT2348	*Norops*	*unilobatus*	18	18	18	40	23	62	21
JHT3352	*Norops*	*unilobatus*	18	18	18	40	23	62	21
JHT3353	*Norops*	*unilobatus*	18	18	18	40	23	61	21
JHT3521	*Norops*	*wellbornae*	18	18	18	45	24	47	22
JHT3522	*Norops*	*wellbornae*	18	18	18	45	24	47	22
JHT3523	*Norops*	*wellbornae*	18	18	18	45	24	47	22
JHT3524	*Norops*	*wellbornae*	18	18	18	45	24	47	22
JHT3555	*Norops*	*wellbornae*	18	18	18	45	24	47	22
JHT3580	*Norops*	*wellbornae*	18	18	18	45	24	47	22
JHT3581	*Norops*	*wellbornae*	18	18	18	45	24	47	22
JHT3597	*Norops*	*wellbornae*	18	18	18	45	24	47	22
JHT3598	*Norops*	*wellbornae*	18	18	18	45	24	47	22
JHT3606	*Norops*	*wellbornae*	18	18	18	45	24	47	22
JHT2355	*Norops*	*rodriguezii*	19	19	19	32	8	63	15
JHT2586	*Norops*	*rodriguezii*	19	19	19	32	8	64	15
JHT3708	*Norops*	*rodriguezii*	19	19	41	37	8	25	16
JHT3709	*Norops*	*rodriguezii*	19	19	41	37	8	25	16
JHT3710	*Norops*	*rodriguezii*	19	19	41	37	8	25	16
JHT3711	*Norops*	*rodriguezii*	19	19	41	37	8	25	16
LDW11424	*Norops*	*rodriguezii*	19	19	41	37	8	25	16
JHT2358	*Norops*	*pijolense*	20	20	20	33	32	17	28
JHT2364	*Norops*	*pijolense*	20	20	20	33	32	17	28
JHT2365	*Norops*	*pijolense*	20	20	20	33	32	17	28
JHT2407	*Norops*	*pijolense*	20	20	20	33	32	17	28
JHT2408	*Norops*	*pijolense*	20	20	20	33	32	17	28
JHT2409	*Norops*	*pijolense*	20	20	20	33	32	17	28
JHT2410	*Norops*	*pijolense*	20	20	20	33	32	17	28

JHT2411	*Norops*	*pijolense*	20	20	20	33	32	17	28
JHT2412	*Norops*	*pijolense*	20	20	20	33	32	17	28
JHT2413	*Norops*	*pijolense*	20	20	20	33	32	17	28
JHT2414	*Norops*	*pijolense*	20	20	20	33	32	17	28
JHT2415	*Norops*	*pijolense*	20	20	20	33	32	17	28
JHT2789	*Norops*	*pijolense*	20	20	20	33	32	17	28
JHT2790	*Norops*	*pijolense*	20	20	20	33	32	17	28
JHT2444	*Norops*	*purpurgularis*	21	21	21	34	31	16	48
JHT2447	*Norops*	*kreutzi*	22	22	22	15	21	18	18
JHT3043	*Norops*	*kreutzi*	22	22	22	15	21	18	18
JHT3103	*Norops*	*kreutzi*	22	22	22	15	21	18	18
JHT2496	*Norops*	*biporcatus*	23	23	23	4	25	30	47
JHT3491	*Norops*	*biporcatus*	23	23	23	4	26	31	26
JHT3497	*Norops*	*biporcatus*	23	23	23	4	26	31	26
N933	*Norops*	*biporcatus*	23	23	23	4	26	31	26
JHT3048	*Norops*	*loveridgei*	24	24	24	24	29	6	29
JHT3049	*Norops*	*loveridgei*	24	24	24	24	29	6	29
JHT3160	*Norops*	*loveridgei*	24	24	24	24	29	6	29
JHT3161	*Norops*	*loveridgei*	24	24	24	24	29	6	29
JHT3270	*Norops*	*loveridgei*	24	24	24	24	29	6	29
MMF205	*Norops*	*loveridgei*	24	24	24	24	29	6	29
MMF207	*Norops*	*loveridgei*	24	24	24	24	29	6	29
JHT2620	*Norops*	*caceresae*	25	25	25	3	35	57	40
JHT2622	*Norops*	*caceresae*	25	25	25	7	35	57	40
JHT2773	*Norops*	*caceresae*	25	25	25	7	35	58	40
JHT2774	*Norops*	*caceresae*	25	25	25	7	35	58	40
JHT2891	*Norops*	*caceresae*	25	25	25	7	35	58	40
JHT2892	*Norops*	*caceresae*	25	25	25	7	35	58	40
JHT2894	*Norops*	*caceresae*	25	25	25	7	35	58	40
JHT2895	*Norops*	*caceresae*	25	25	25	7	35	58	40
JHT3742	*Norops*	*caceresae*	25	25	25	7	35	58	40

JHT3743	*Norops*	*caceresae*	25	25	25	7	35	58	40
JHT3764	*Norops*	*caceresae*	25	25	25	7	35	58	40
JHT3765	*Norops*	*caceresae*	25	25	25	7	35	58	40
JHT3766	*Norops*	*caceresae*	25	25	25	7	35	58	40
JHT3767	*Norops*	*caceresae*	25	25	25	7	35	58	40
JHT3768	*Norops*	*caceresae*	25	25	25	7	35	58	40
JHT3769	*Norops*	*caceresae*	25	25	25	7	35	58	40
JHT3770	*Norops*	*caceresae*	25	25	25	7	35	58	40
JHT3781	*Norops*	*caceresae*	25	25	25	7	35	58	40
JHT3822	*Norops*	*caceresae*	25	25	25	7	35	58	40
JHT3887	*Norops*	*caceresae*	25	25	25	7	35	58	40
JHT3888	*Norops*	*caceresae*	25	25	25	7	35	58	40
JHT2623	*Norops*	*rubribarbaris (aff)*	26	26	26	35	37	12	39
JHT2876	*Norops*	*rubribarbaris (aff)*	26	26	26	35	37	12	39
JHT2845	*Norops*	*oscelloscapularis*	27	27	27	27	15	10	43
JHT3276	*Anolis*	*allisoni*	28	28	28	1	1	1	23
JHT3292	*Anolis*	*allisoni*	28	28	28	1	1	1	23
JHT3423	*Norops*	*cupreus*	29	29	29	47	3	37	15
JHT3466	*Norops*	*cupreus*	29	29	29	47	3	37	15
JHT3467	*Norops*	*cupreus*	29	29	29	47	3	37	15
JMS71	*Norops*	*cupreus*	29	29	29	8	3	35	41
MMF107	*Norops*	*cupreus*	29	29	29	9	3	39	15
MMF108	*Norops*	*cupreus*	29	29	29	9	3	54	15
MMF109	*Norops*	*cupreus*	29	29	29	9	3	59	15
MMF110	*Norops*	*cupreus*	29	29	29	9	3	60	15
MMF111	*Norops*	*cupreus*	29	29	29	9	3	44	15
MMF112	*Norops*	*cupreus*	29	29	29	9	3	43	15
MMF114	*Norops*	*cupreus*	29	29	29	9	3	41	15
MMF115	*Norops*	*cupreus*	29	29	29	9	3	42	15
MMF168	*Norops*	*cupreus*	29	29	29	9	3	38	15
MMF169	*Norops*	*cupreus*	29	29	29	9	3	40	15

N198	*Norops*	*cupreus*	29	29	29	51	3	36	15
N674	*Norops*	*cupreus*	29	29	29	51	3	36	15
KU289793	*Norops*	*crassulus*	30	30	30	6	34	8	50
KU291367	*Norops*	*serranoi*	31	31	31	36	12	14	44
N087	*Norops*	*oxylophus*	32	32	32	30	11	7	12
N590	*Norops*	*oxylophus*	32	32	32	30	11	7	12
N608	*Norops*	*oxylophus*	32	32	32	30	11	7	12
N562	*Norops*	*wermuthi*	33	33	33	46	43	56	34
N956	*Norops*	*wermuthi*	33	33	33	46	43	55	34
GK5192	*Norops*	*uniformis*	34	34	34	42	27	32	24
GK5228	*Norops*	*uniformis*	34	34	42	39	28	33	25
GK5229	*Norops*	*uniformis*	34	34	42	42	28	33	25
GK5316	*Norops*	*uniformis*	34	34	42	42	28	33	25
GK5538	*Norops*	*uniformis*	34	34	34	39	27	32	24

Supplementary Table 4. Assignment of specimens to clusters (given as cluster number) based on all analyses of the reduced dataset (290 sequences). Specimens are labelled with either Field or Museum IDs.

ID	*Genus*	*species*	ABGD 7.2%	ABGD 5.2%	BOLD RESL	mPTP	bPTP	GMYC
JHT3276	*Anolis*	*allisoni*	1	1	1	1	1	25
JHT3292	*Anolis*	*allisoni*	1	1	1	1	1	25
JHT1596	*Norops*	*amplisquamosus*	3	3	2	2	3	31
JHT1615	*Norops*	*amplisquamosus*	3	3	2	2	3	31
JHT1620	*Norops*	*amplisquamosus*	3	3	2	2	3	31
JHT1621	*Norops*	*amplisquamosus*	3	3	2	2	3	31
JHT2986	*Norops*	*amplisquamosus*	3	3	2	2	3	31
JHT2496	*Norops*	*biporcatus*	13	13	3	3	31	48
JHT3491	*Norops*	*biporcatus*	13	13	4	4	32	24
JHT3497	*Norops*	*biporcatus*	13	13	4	4	32	24
N933	*Norops*	*biporcatus*	13	13	4	4	32	24
JHT2620	*Norops*	*caceresae*	12	12	7	7	53	32
JHT2622	*Norops*	*caceresae*	12	12	7	7	53	32
JHT2773	*Norops*	*caceresae*	12	12	7	7	54	32
JHT2774	*Norops*	*caceresae*	12	12	7	7	54	32
JHT2891	*Norops*	*caceresae*	12	12	7	7	54	32
JHT2892	*Norops*	*caceresae*	12	12	7	7	54	32
JHT2895	*Norops*	*caceresae*	12	12	7	7	54	32
JHT3742	*Norops*	*caceresae*	12	12	7	7	54	32
JHT3743	*Norops*	*caceresae*	12	12	7	7	54	32
JHT3767	*Norops*	*caceresae*	12	12	7	7	54	32
JHT3822	*Norops*	*caceresae*	12	12	7	7	54	32
JHT2266	*Norops*	*capito*	14	14	5	5	9	5
JHT2267	*Norops*	*capito*	14	14	5	5	9	5
JHT3416	*Norops*	*capito*	14	14	5	5	9	5
MMF113	*Norops*	*capito*	14	14	5	5	9	5

MMF133	*Norops*	*capito*	14	14	5	5	9	5
N1016	*Norops*	*capito*	14	14	5	5	9	5
N117	*Norops*	*capito*	14	14	5	5	9	5
N266	*Norops*	*capito*	14	14	5	5	9	5
KU289793	*Norops*	*crassulus*	11	11	6	6	8	50
JHT3423	*Norops*	*cupreus*	18	18	47	8	44	7
JHT3466	*Norops*	*cupreus*	18	18	47	8	44	7
JHT3467	*Norops*	*cupreus*	18	18	47	8	44	7
JMS71	*Norops*	*cupreus*	18	18	8	8	45	42
MMF107	*Norops*	*cupreus*	18	18	9	8	42	8
MMF108	*Norops*	*cupreus*	18	18	9	8	40	8
MMF109	*Norops*	*cupreus*	18	18	9	8	59	8
MMF111	*Norops*	*cupreus*	18	18	9	8	41	8
MMF168	*Norops*	*cupreus*	18	18	9	8	43	8
N198	*Norops*	*cupreus*	18	18	51	8	46	7
N674	*Norops*	*cupreus*	18	18	51	8	46	7
JHT1301	*Norops*	*cusuco*	27	27	10	9	19	18
JHT2983	*Norops*	*cusuco*	27	27	10	9	19	18
JHT2985	*Norops*	*cusuco*	27	27	10	9	19	18
JHT2317	*Norops*	*heteropholidotus*	6	6	13	12	39	37
JHT2318	*Norops*	*heteropholidotus*	6	6	13	12	39	37
JHT2321	*Norops*	*heteropholidotus*	6	6	13	12	39	37
JHT2322	*Norops*	*heteropholidotus*	6	6	13	12	39	37
JHT2323	*Norops*	*heteropholidotus*	6	6	13	12	39	37
JHT2702	*Norops*	*heteropholidotus*	6	6	12	11	36	38
JHT2703	*Norops*	*heteropholidotus*	6	6	12	11	36	38
JHT2706	*Norops*	*heteropholidotus*	6	6	12	11	36	38
JHT2707	*Norops*	*heteropholidotus*	6	6	12	11	36	38
JHT2721	*Norops*	*heteropholidotus*	6	6	12	11	36	38
JHT2723	*Norops*	*heteropholidotus*	6	6	12	11	36	38
JHT2885	*Norops*	*heteropholidotus*	6	6	13	12	39	37

JHT2893	*Norops*	*heteropholidotus*	6	6	13	12	39	37
JHT3639	*Norops*	*heteropholidotus*	6	6	14	13	38	51
JHT3763	*Norops*	*heteropholidotus*	6	6	13	12	39	37
JHT3817	*Norops*	*heteropholidotus*	6	6	13	12	39	37
JHT3818	*Norops*	*heteropholidotus*	6	6	13	12	39	37
JHT3892	*Norops*	*heteropholidotus*	6	6	13	12	39	37
JHT3903	*Norops*	*heteropholidotus*	6	6	13	12	39	37
KU291251	*Norops*	*heteropholidotus*	6	6	12	11	36	38
MMF005	*Norops*	*heteropholidotus*	6	6	11	10	35	39
MMF006	*Norops*	*heteropholidotus*	6	6	11	10	35	39
JHT1586	*Norops*	*johnmeyeri*	32	32	16	39	5	26
JHT1587	*Norops*	*johnmeyeri*	32	32	16	39	5	26
JHT1594	*Norops*	*johnmeyeri*	32	32	16	39	5	26
JHT1606	*Norops*	*johnmeyeri*	32	32	16	39	5	26
JHT1607	*Norops*	*johnmeyeri*	32	32	16	39	5	26
JHT2447	*Norops*	*kreutzi*	28	28	15	33	18	19
JHT3043	*Norops*	*kreutzi*	28	28	15	33	18	19
JHT2000	*Norops*	*laeviventris*	30	30	18	36	48	20
JHT2152	*Norops*	*laeviventris*	30	30	18	36	48	20
JHT2229	*Norops*	*laeviventris*	30	30	18	36	48	20
JHT2278	*Norops*	*laeviventris*	30	30	17	36	47	21
JHT2284	*Norops*	*laeviventris*	30	30	17	36	47	21
JHT2320	*Norops*	*laeviventris*	30	30	18	36	48	20
JHT2394	*Norops*	*laeviventris*	30	30	37	37	33	46
JHT2532	*Norops*	*laeviventris*	30	30	18	36	48	20
JHT2533	*Norops*	*laeviventris*	30	30	18	36	48	20
JHT2542	*Norops*	*laeviventris*	30	30	18	36	48	20
JHT2545	*Norops*	*laeviventris*	30	30	18	36	48	20
JHT2973	*Norops*	*laeviventris*	30	30	18	36	48	20
JHT3390	*Norops*	*laeviventris*	30	30	18	36	48	20
JHT3635	*Norops*	*laeviventris*	30	30	18	36	48	20

JHT3916	*Norops*	*laeviventris*	30	30	18	36	48	20
JHT4005	*Norops*	*laeviventris*	30	30	17	36	47	21
MMF170	*Norops*	*laeviventris*	30	30	18	36	48	20
MMF171	*Norops*	*laeviventris*	30	30	18	36	48	20
CAC025	*Norops*	*lemurinus*	24	24	19	28	49	11
IRL022	*Norops*	*lemurinus*	24	24	20	29	50	12
IRL032	*Norops*	*lemurinus*	24	24	20	29	50	12
JHT2346	*Norops*	*lemurinus*	24	24	20	29	50	12
JHT2347	*Norops*	*lemurinus*	24	24	20	29	50	12
JHT2383	*Norops*	*lemurinus*	24	24	20	29	50	12
JHT2484	*Norops*	*lemurinus*	24	24	20	29	50	12
JHT2491	*Norops*	*lemurinus*	24	24	20	29	50	12
JHT2495	*Norops*	*lemurinus*	24	24	20	29	50	12
JHT2500	*Norops*	*lemurinus*	24	24	20	29	50	12
JHT2585	*Norops*	*lemurinus*	24	24	20	29	50	12
JHT3023	*Norops*	*lemurinus*	24	24	20	29	50	12
JHT3203	*Norops*	*lemurinus*	24	24	20	29	50	12
JHT3204	*Norops*	*lemurinus*	24	24	20	29	50	12
JHT3291	*Norops*	*lemurinus*	24	24	20	29	50	12
JHT3422	*Norops*	*lemurinus*	24	24	19	28	49	11
JHT3488	*Norops*	*limifrons*	25	25	21	31	27	15
JHT3959	*Norops*	*limifrons*	25	25	21	31	27	15
JHT3960	*Norops*	*limifrons*	25	25	21	31	27	15
JHT3965	*Norops*	*limifrons*	25	25	21	31	27	15
JHT3979	*Norops*	*limifrons*	25	25	21	31	27	15
JHT3980	*Norops*	*limifrons*	25	25	21	31	27	15
JHT3982	*Norops*	*limifrons*	25	25	21	31	27	15
JHT3983	*Norops*	*limifrons*	25	25	21	31	27	15
JHT2576	*Norops*	*loveridgei*	31	31	24	38	6	28
JHT3048	*Norops*	*loveridgei*	31	31	24	38	6	28
JHT3160	*Norops*	*loveridgei*	31	31	24	38	6	28

JHT3161	*Norops*	*loveridgei*	31	31	24	38	6	28
JHT3270	*Norops*	*loveridgei*	31	31	24	38	6	28
MMF205	*Norops*	*loveridgei*	31	31	24	38	6	28
MMF207	*Norops*	*loveridgei*	31	31	24	38	6	28
CAC005	*Norops*	*mccraniei*	36	37	23	19	22	1
CAC043	*Norops*	*mccraniei*	36	37	23	19	22	2
JHT1402	*Norops*	*mccraniei*	36	37	23	19	22	1
JHT1403	*Norops*	*mccraniei*	36	37	23	19	22	1
JHT1404	*Norops*	*mccraniei*	36	37	23	19	22	1
JHT1412	*Norops*	*mccraniei*	36	37	23	19	22	1
JHT1414	*Norops*	*mccraniei*	36	37	23	19	22	1
JHT1699	*Norops*	*mccraniei*	36	37	23	19	22	1
JHT2037	*Norops*	*mccraniei*	36	37	23	19	22	2
JHT2038	*Norops*	*mccraniei*	36	37	23	19	22	2
JHT2127	*Norops*	*mccraniei*	36	37	23	19	22	2
JHT2147	*Norops*	*mccraniei*	36	37	23	19	22	1
JHT2303	*Norops*	*mccraniei*	36	37	23	19	22	2
JHT2354	*Norops*	*mccraniei*	36	37	23	19	22	2
JHT2424	*Norops*	*mccraniei*	36	37	23	19	22	1
JHT2425	*Norops*	*mccraniei*	36	37	23	19	22	1
JHT2460	*Norops*	*mccraniei*	36	37	23	19	22	1
JHT2462	*Norops*	*mccraniei*	36	37	23	19	22	1
JHT2962	*Norops*	*mccraniei*	36	37	23	19	22	2
JHT3006	*Norops*	*mccraniei*	36	37	23	19	22	2
JHT3365	*Norops*	*mccraniei*	36	37	23	19	22	1
JHT3366	*Norops*	*mccraniei*	36	37	23	19	22	1
JHT3367	*Norops*	*mccraniei*	36	37	23	19	22	1
JHT3393	*Norops*	*mccraniei*	36	37	23	19	22	1
JHT3394	*Norops*	*mccraniei*	36	37	23	19	22	1
JHT3395	*Norops*	*mccraniei*	36	37	23	19	22	1
JHT3414	*Norops*	*mccraniei*	36	37	23	19	22	1

JHT3415	*Norops*	*mccraniei*	36	37	23	19	22	1
JHT3930	*Norops*	*mccraniei*	36	37	23	19	22	1
MMF132	*Norops*	*mccraniei*	36	37	23	19	22	1
MMF148	*Norops*	*mccraniei*	36	37	23	19	22	1
JHT3633	*Norops*	*mccraniei (aff)*	16	16	26	20	21	3
JHT3634	*Norops*	*mccraniei (aff)*	16	16	26	20	21	3
JHT3652	*Norops*	*mccraniei (aff)*	16	16	26	20	21	3
JHT3653	*Norops*	*mccraniei (aff)*	16	16	26	20	21	3
JHT3663	*Norops*	*mccraniei (aff)*	16	16	26	20	21	3
JHT3943	*Norops*	*mccraniei (aff)*	16	16	26	20	21	3
JHT3944	*Norops*	*mccraniei (aff)*	16	16	26	20	21	3
JHT1638	*Norops*	*morazani*	5	5	25	45	24	33
JHT1639	*Norops*	*morazani*	5	5	25	45	24	33
JHT2040	*Norops*	*morazani*	5	5	25	45	24	33
JHT2041	*Norops*	*morazani*	5	5	25	45	24	33
JHT2042	*Norops*	*morazani*	5	5	25	45	24	33
JHT2049	*Norops*	*morazani*	5	5	25	45	24	33
JHT2051	*Norops*	*morazani*	5	5	25	45	24	33
JHT2052	*Norops*	*morazani*	5	5	25	45	24	33
JHT2053	*Norops*	*morazani*	5	5	25	45	24	33
JHT2054	*Norops*	*morazani*	5	5	25	45	24	33
JHT2056	*Norops*	*morazani*	5	5	25	45	24	33
JHT2080	*Norops*	*morazani*	5	5	25	45	24	33
JHT2095	*Norops*	*morazani*	5	5	25	45	24	33
JHT2096	*Norops*	*morazani*	5	5	25	45	24	33
JHT2097	*Norops*	*morazani*	5	5	25	45	24	33
JHT2098	*Norops*	*morazani*	5	5	25	45	24	33
JHT2102	*Norops*	*morazani*	5	5	25	45	24	33
JHT2109	*Norops*	*morazani*	5	5	25	45	24	33
JHT2111	*Norops*	*morazani*	5	5	25	45	24	33
JHT2967	*Norops*	*morazani*	5	5	25	45	24	33

JHT2969	*Norops*	*morazani*	5	5	25	45	24	33
JHT2970	*Norops*	*morazani*	5	5	25	45	24	33
JHT2971	*Norops*	*morazani*	5	5	25	45	24	33
JHT3188	*Norops*	*morazani (aff)*	35	36	28	46	23	34
JHT3189	*Norops*	*morazani (aff)*	35	36	28	46	23	34
JHT3190	*Norops*	*morazani (aff)*	35	36	28	46	23	34
JHT2845	*Norops*	*ocelloscapularis*	20	20	27	25	10	44
N087	*Norops*	*oxylophus*	22	22	30	26	7	13
N590	*Norops*	*oxylophus*	22	22	30	26	7	13
JHT1499	*Norops*	*petersii*	4	4	29	44	4	47
JHT2358	*Norops*	*pijolensis*	34	34	33	41	17	27
JHT2364	*Norops*	*pijolensis*	34	34	33	41	17	27
JHT2408	*Norops*	*pijolensis*	34	34	33	41	17	27
JHT2409	*Norops*	*pijolensis*	34	34	33	41	17	27
JHT2411	*Norops*	*pijolensis*	34	34	33	41	17	27
JHT2413	*Norops*	*pijolensis*	34	34	33	41	17	27
JHT2789	*Norops*	*pijolensis*	34	34	33	41	17	27
JHT2444	*Norops*	*purpurgularis*	33	33	34	40	16	49
JHT2281	*Norops*	*quaggulus*	17	17	31	21	2	6
JHT2282	*Norops*	*quaggulus*	17	17	31	21	2	6
N273	*Norops*	*quaggulus*	17	17	31	21	2	6
JHT2355	*Norops*	*rodriguezii*	26	26	32	32	58	16
JHT2586	*Norops*	*rodriguezii*	26	26	32	32	25	16
JHT3708	*Norops*	*rodriguezii*	26	38	35	32	26	17
JHT3711	*Norops*	*rodriguezii*	26	38	35	32	26	17
LDW11424	*Norops*	*rodriguezii*	26	38	35	32	26	17
JHT2342	*Norops*	*rubribarbaris*	10	10	38	16	13	40
JHT2343	*Norops*	*rubribarbaris*	10	10	38	16	13	40
JHT2344	*Norops*	*rubribarbaris*	10	10	38	16	13	40
JHT3224	*Norops*	*rubribarbaris*	10	10	38	16	13	40
JHT3323	*Norops*	*rubribarbaris*	10	10	38	16	13	40

JHT2623	*Norops*	*rubribarbaris (aff)*	9	9	35	17	15	41
JHT2876	*Norops*	*rubribarbaris (aff)*	9	9	35	17	12	41
KU291367	*Norops*	*serranoi*	23	23	36	27	14	45
JHT2289	*Norops*	*sminthus*	8	8	41	15	15	35
JHT2290	*Norops*	*sminthus*	8	8	41	15	15	35
JHT2292	*Norops*	*sminthus*	8	8	41	15	15	35
JHT2295	*Norops*	*sminthus*	8	8	41	15	15	35
JHT2296	*Norops*	*sminthus*	8	8	41	15	15	35
JHT2297	*Norops*	*sminthus*	8	8	41	15	15	35
JHT2298	*Norops*	*sminthus*	8	8	41	15	15	35
JHT2302	*Norops*	*sminthus*	8	8	41	15	15	35
SMF100139	*Norops*	*uniformis*	2	2	39	42	51	29
SMF99539	*Norops*	*uniformis*	2	2	39	42	52	29
SMF99541	*Norops*	*uniformis*	2	35	42	43	34	30
SMF99542	*Norops*	*uniformis*	2	35	42	43	34	30
SMF99546	*Norops*	*uniformis*	2	35	42	43	34	30
JHT2348	*Norops*	*unilobatus*	29	29	40	35	56	22
JHT3352	*Norops*	*unilobatus*	29	29	40	35	56	22
JHT3353	*Norops*	*unilobatus*	29	29	40	35	55	22
JHT3521	*Norops*	*wellbornae*	29	29	45	34	37	23
JHT3522	*Norops*	*wellbornae*	29	29	45	34	37	23
JHT3523	*Norops*	*wellbornae*	29	29	45	34	37	23
JHT3524	*Norops*	*wellbornae*	29	29	45	34	37	23
JHT3555	*Norops*	*wellbornae*	29	29	45	34	37	23
JHT3580	*Norops*	*wellbornae*	29	29	45	34	37	23
JHT3581	*Norops*	*wellbornae*	29	29	45	34	37	23
JHT3597	*Norops*	*wellbornae*	29	29	45	34	37	23
JHT3606	*Norops*	*wellbornae*	29	29	45	34	37	23
N562	*Norops*	*wermuthi*	7	7	46	14	57	36
N956	*Norops*	*wermuthi*	7	7	46	14	57	36
CAC048	*Norops*	*wilsoni*	15	15	43	18	20	4

CAC050	*Norops*	*wilsoni*	15	15	43	18	20	4
JHT3361	*Norops*	*wilsoni*	15	15	43	18	20	4
MMF204	*Norops*	*wilsoni*	15	15	43	18	20	4
JHT1415	*Norops*	*yoroensis*	19	19	49	24	29	43
JHT2032	*Norops*	*yoroensis*	19	19	44	22	30	10
JHT2367	*Norops*	*yoroensis*	19	19	44	22	30	10
JHT2368	*Norops*	*yoroensis*	19	19	44	22	30	10
JHT2391	*Norops*	*yoroensis*	19	19	44	22	30	10
JHT2393	*Norops*	*yoroensis*	19	19	44	22	30	10
JHT2400	*Norops*	*yoroensis*	19	19	44	22	30	10
JHT2402	*Norops*	*yoroensis*	19	19	44	22	30	10
JHT2403	*Norops*	*yoroensis*	19	19	44	22	30	10
JHT2404	*Norops*	*yoroensis*	19	19	44	22	30	10
JHT2784	*Norops*	*yoroensis*	19	19	44	22	30	10
JHT2792	*Norops*	*yoroensis*	19	19	44	22	30	10
JHT2942	*Norops*	*yoroensis*	19	19	44	22	30	10
JHT2943	*Norops*	*yoroensis*	19	19	44	22	30	10
JHT2944	*Norops*	*yoroensis*	19	19	44	22	30	10
CAC011	*Norops*	*yoroensis*	21	21	50	23	11	9
JHT3044	*Norops*	*yoroensis*	21	21	50	23	11	9
JHT3052	*Norops*	*yoroensis*	21	21	50	23	11	9
JHT3086	*Norops*	*yoroensis*	21	21	50	23	11	9
JHT3088	*Norops*	*yoroensis*	21	21	50	23	11	9
JHT3092	*Norops*	*yoroensis*	21	21	50	23	11	9
JHT3093	*Norops*	*yoroensis*	21	21	50	23	11	9
JHT3140	*Norops*	*yoroensis*	21	21	50	23	11	9
JHT3162	*Norops*	*yoroensis*	21	21	50	23	11	9
JHT3163	*Norops*	*yoroensis*	21	21	50	23	11	9
JHT3249	*Norops*	*yoroensis*	21	21	50	23	11	9
LDW13136	*Norops*	*yoroensis*	21	21	50	23	11	9
MMF198	*Norops*	*yoroensis*	21	21	50	23	11	9

MMF199	*Norops*	*yoroensis*	21	21	50	23	11	9
MMF200	*Norops*	*yoroensis*	21	21	50	23	11	9
MMF202	*Norops*	*yoroensis*	21	21	50	23	11	9
MMF206	*Norops*	*yoroensis*	21	21	50	23	11	9
IRL061	*Norops*	*zeus*	25	25	48	30	28	14
JHT2378	*Norops*	*zeus*	25	25	48	30	28	14
JHT2379	*Norops*	*zeus*	25	25	48	30	28	14
JHT2381	*Norops*	*zeus*	25	25	48	30	28	14
JHT2515	*Norops*	*zeus*	25	25	48	30	28	14
JHT2981	*Norops*	*zeus*	25	25	48	30	28	14
JHT2982	*Norops*	*zeus*	25	25	48	30	28	14
JHT3025	*Norops*	*zeus*	25	25	48	30	28	14
JHT3036	*Norops*	*zeus*	25	25	48	30	28	14
JHT3343	*Norops*	*zeus*	25	25	48	30	28	14
JHT3344	*Norops*	*zeus*	25	25	48	30	28	14
JHT3359	*Norops*	*zeus*	25	25	48	30	28	14

1.2 Supplementary Figures

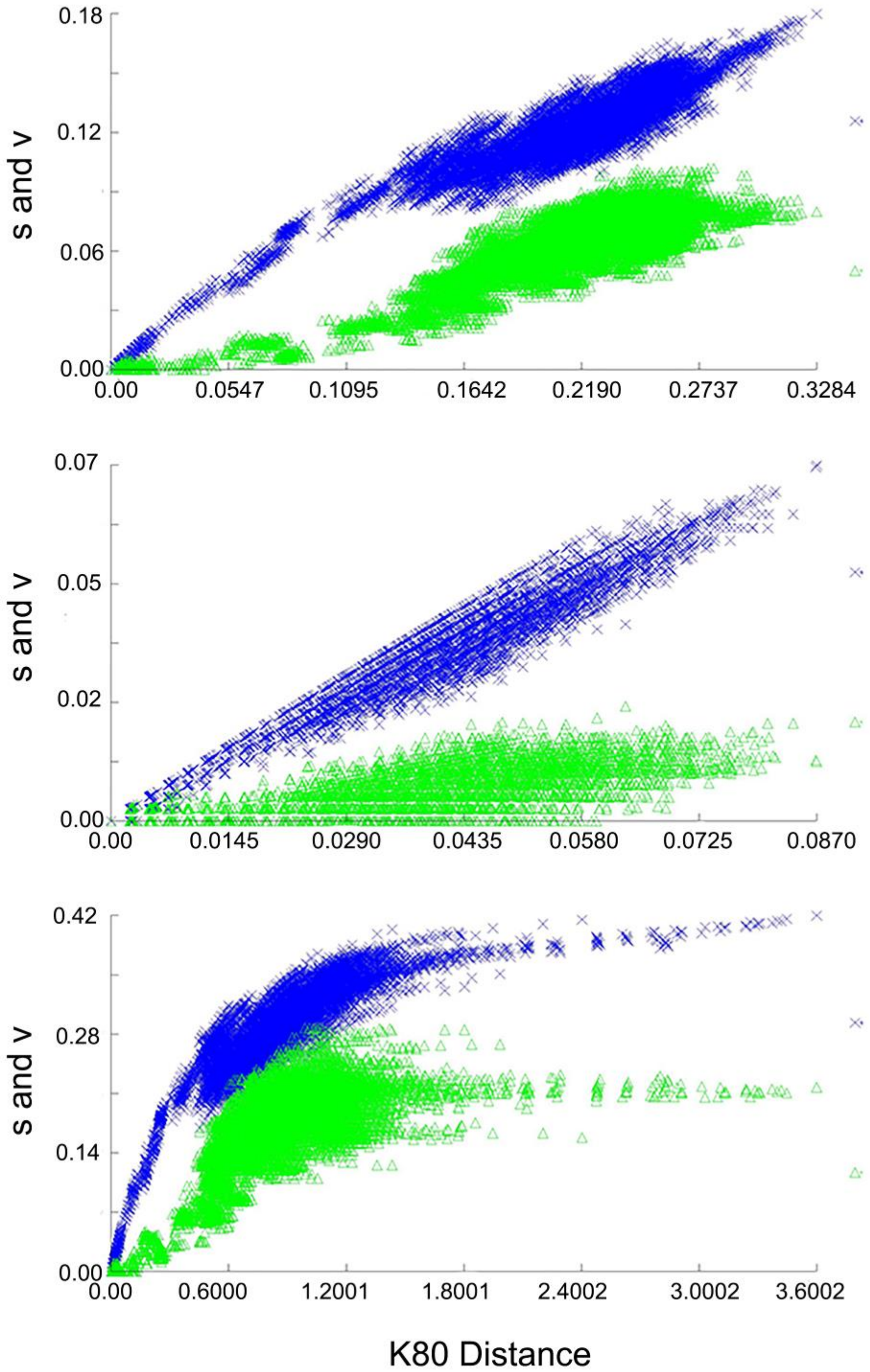

Supplementary Figure 1. Plots of transitions ("s"/×; blue) and transversions ("v"/Δ; green) vs. K2P distance: all codon positions (top), 1st & 2nd codon position (middle), and 3rd codon position only (bottom).

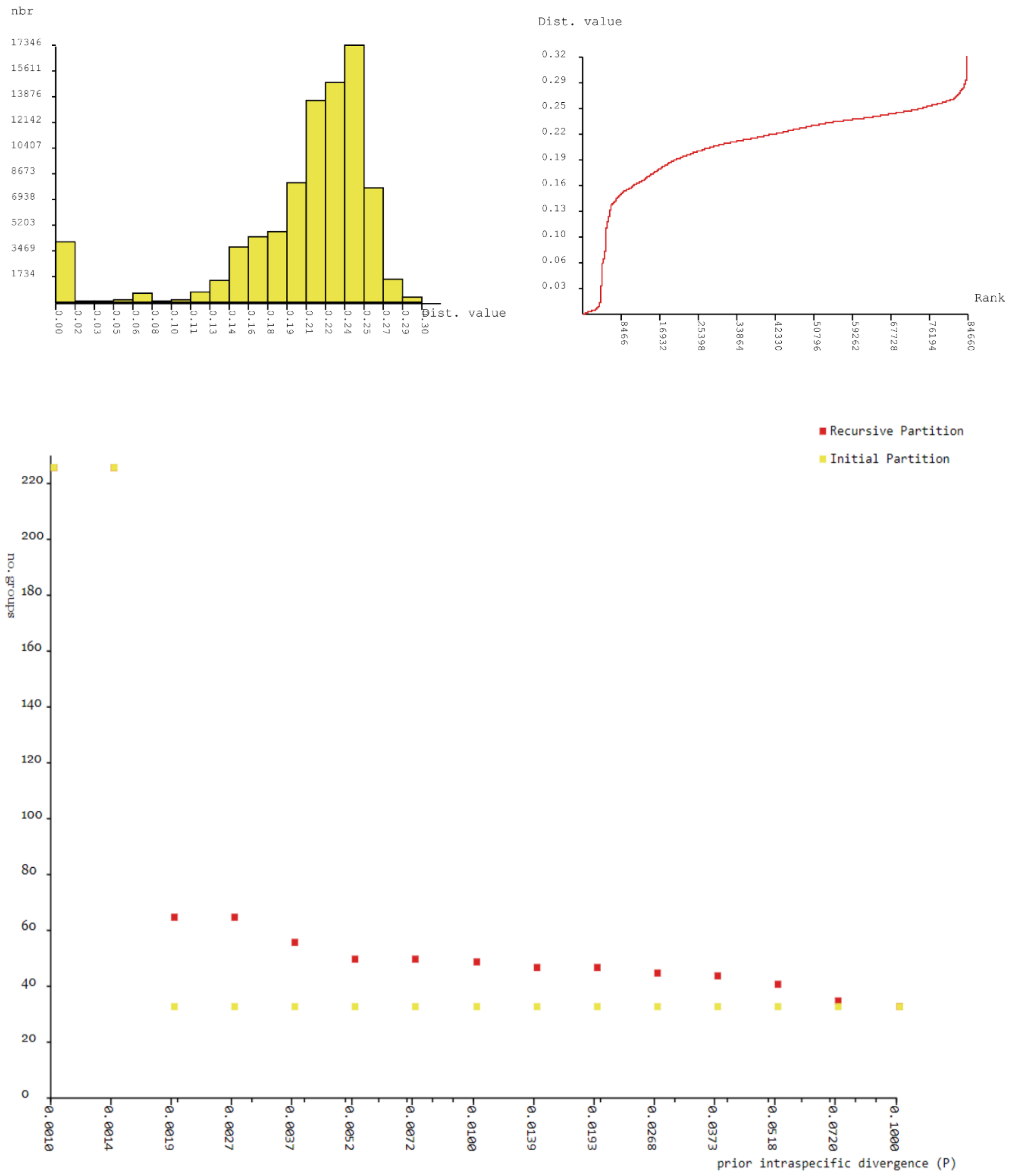

Supplementary Figure 2. Output of ABGD analyses on the full dataset.

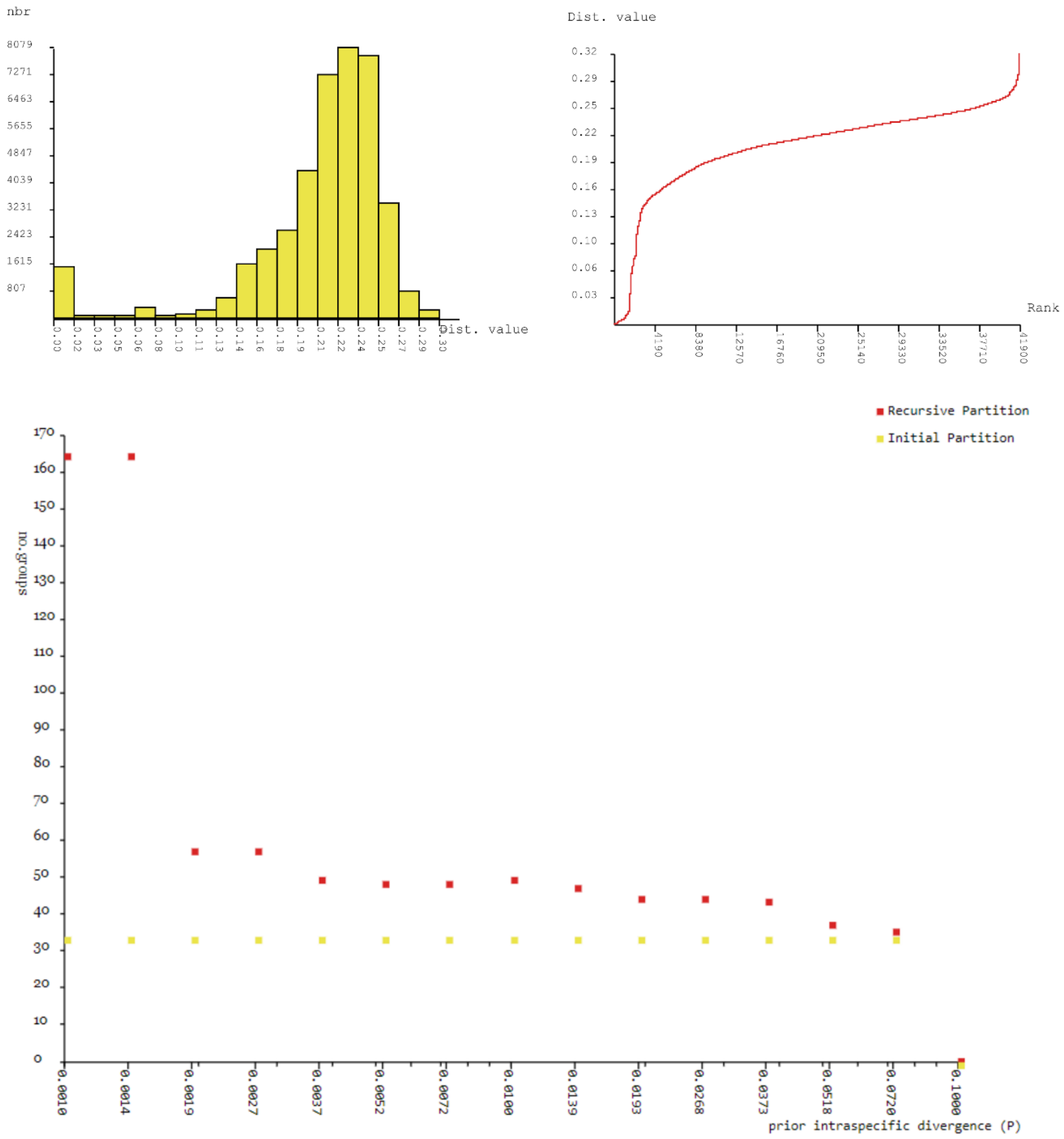

Supplementary Figure 3. Output of ABGD analyses on the reduced dataset.

2 Supplementary Data Sheet

2.1 Supplementary Data Sheet 1.

Full distance data (uncorrected p, K2P, and TrN+G) for samples as assigned *a priori* and by species delimitation methods (ABGD at 7.2% threshold and mPTP).

Attached - "SupplementaryDataSheet2.1_alldistancedata.xlsx"
